# The chemerin/CMKLR1 axis regulates intestinal graft-versus-host disease

**DOI:** 10.1172/jci.insight.154440

**Published:** 2023-03-08

**Authors:** Erica Dander, Paola Vinci, Stefania Vetrano, Camilla Recordati, Rocco Piazza, Grazia Fazio, Donatella Bardelli, Mattia Bugatti, Francesca Sozio, Andrea Piontini, Sonia Bonanomi, Luca Bertola, Elena Tassistro, Maria Grazia Valsecchi, Stefano Calza, William Vermi, Andrea Biondi, Annalisa Del Prete, Silvano Sozzani, Giovanna D’Amico

**Affiliations:** 1 Tettamanti Center, Fondazione IRCCS San Gerardo dei Tintori, Monza, Italy.; 2 Laboratory of Gastrointestinal Immunopathology, Humanitas Clinical and Research Center, Rozzano, Italy.; 3 Department of Biomedical Sciences, Humanitas University, Pieve Emanuele, Italy.; 4 Department of Veterinary Medicine, University of Milan, Lodi, Italy.; 5 Mouse and Animal Pathology Laboratory, Fondazione Unimi, Milan, Italy.; 6 Department of Medicine and Surgery, University of Milano-Bicocca, Monza, Italy.; 7 Hematology Division and Bone Marrow Unit, San Gerardo Hospital, Monza, Italy.; 8 Department of Molecular and Translational Medicine, University of Brescia, Brescia, Italy.; 9 Humanitas Clinical and Research Center–IRCCS, Rozzano, Italy.; 10 Pediatrics, Fondazione IRCCS San Gerardo dei Tintori, Monza, Italy.; 11 Bicocca Center of Bioinformatics, Biostatistics and Bioimaging (B4 center), School of Medicine and Surgery, University of Milano-Bicocca, Monza, Italy.; 12 Biostatistics, Department of Molecular and Translational Medicine, University of Brescia, Brescia, Italy.; 13 School of Medicine and Surgery, University of Milano-Bicocca, Monza, Italy.; 14 Department of Molecular Medicine, Sapienza University of Rome, Laboratory affiliated to Istituto Pasteur Italia – Fondazione Cenci Bolognetti, Rome, Italy.; 15 IRCCS Neuromed, Pozzilli, Italy.

**Keywords:** Immunology, Transplantation, Bone marrow transplantation, Chemokines, Macrophages

## Abstract

Gastrointestinal graft-versus-host disease (GvHD) is a major cause of mortality and morbidity following allogeneic bone marrow transplantation (allo-BMT). Chemerin is a chemotactic protein that recruits leukocytes to inflamed tissues by interacting with ChemR23/CMKLR1, a chemotactic receptor expressed by leukocytes, including macrophages. During acute GvHD, chemerin plasma levels were strongly increased in allo-BM-transplanted mice. The role of the chemerin/CMKLR1 axis in GvHD was investigated using *Cmklr1*-KO mice. WT mice transplanted with an allogeneic graft from *Cmklr1*-KO donors (t-KO) had worse survival and more severe GvHD. Histological analysis demonstrated that the gastrointestinal tract was the organ mostly affected by GvHD in t-KO mice. The severe colitis of t-KO mice was characterized by massive neutrophil infiltration and tissue damage associated with bacterial translocation and exacerbated inflammation. Similarly, *Cmklr1*-KO recipient mice showed increased intestinal pathology in both allogeneic transplant and dextran sulfate sodium–induced colitis. Notably, the adoptive transfer of WT monocytes into t-KO mice mitigated GvHD manifestations by decreasing gut inflammation and T cell activation. In patients, higher chemerin serum levels were predictive of GvHD development. Overall, these results suggest that CMKLR1/chemerin may be a protective pathway for the control of intestinal inflammation and tissue damage in GvHD.

## Graphical abstract

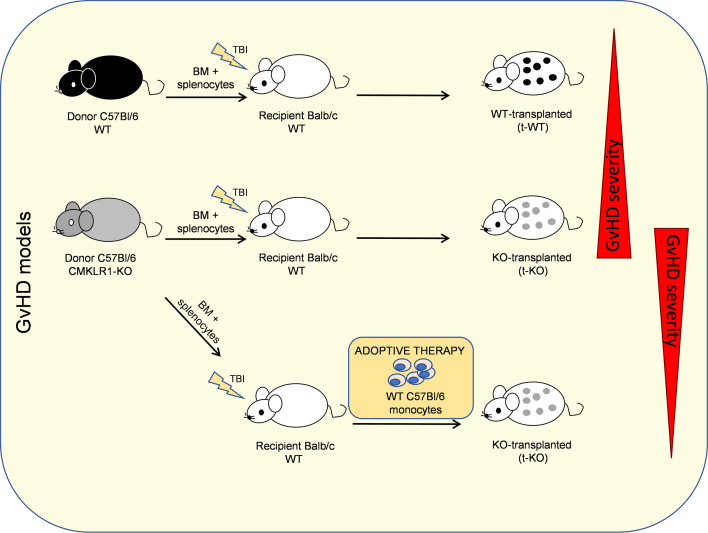

## Introduction

Acute gastrointestinal (GI) graft-versus-host disease (GvHD) is one of the leading causes of transplant-related mortality after allogeneic hematopoietic stem cell transplantation, affecting up to 60% of patients. Diagnosis of this potentially fatal disease presents significant challenges, including the fact that its manifestations are often clinically indistinguishable from other causes of GI dysfunction and often require confirmatory biopsies. The use of steroids as first-line treatment is not always effective and is associated with increased risk of infections and relapse, as well as other side effects (1, 2). Thus, new biomarkers are needed to guide targeted therapies.

The pathophysiology of acute GvHD occurs in 3 phases. First, the conditioning regimen administered prior to transplant induces severe tissue damage in recipients, resulting in host antigen-presenting cell (APC) activation. Second, activated APCs produce several proinflammatory cytokines and chemokines that stimulate donor T cell activation. Finally, in the effector phase, infiltration of activated T cells, macrophages, natural killer cells, and cytokines damages GvHD target organs (3, 4). Although the role of professional APCs and T cells in GvHD pathophysiology is well established, other immune cell populations have been more recently implicated, including myeloid cells (5–7). The trafficking of proinflammatory and antiinflammatory immune effector cells to GvHD target organs is orchestrated by chemotactic factors (8–10). Chemerin is a chemotactic protein that has been implicated in both the promotion and the resolution of the inflammatory response (11–16). Chemerin is secreted as a precursor protein that becomes active after processing at the C-terminus by several cysteine and serine proteases, such as those involved in coagulation, fibrinolysis, and inflammation (17, 18). Active chemerin induces the migration of cells expressing the G protein–coupled receptor CMKLR1, such as immature myeloid dendritic cells (14, 19), plasmacytoid dendritic cells (15), macrophages (20), and natural killer cells (21). The chemerin/CMKLR1 axis was implicated in the pathophysiology of various autoimmune diseases, such as systemic lupus erythematosus (15, 22), psoriasis (23, 24), rheumatoid arthritis (25), and tumors (26–29).

This study investigated the role of the chemerin/CMKLR1 axis in GvHD, using a well-known MHC-mismatched murine model (30) based on the use of BALB/c (H-2^d^) as recipient mice and C57BL/6 (B6; H-2^b^) mice as allograft donors.

Results here presented support a role for chemerin/CMKLR1 in the modulation of intestinal inflammation and GvHD tissue damage. Furthermore, we demonstrated that CMKLR1^+^ monocytes mitigated GvHD manifestations by decreasing gut inflammation and T cell activation.

## Results

### Role of the chemerin/CMKLR1 axis in acute GvHD.

**Figure 1:**
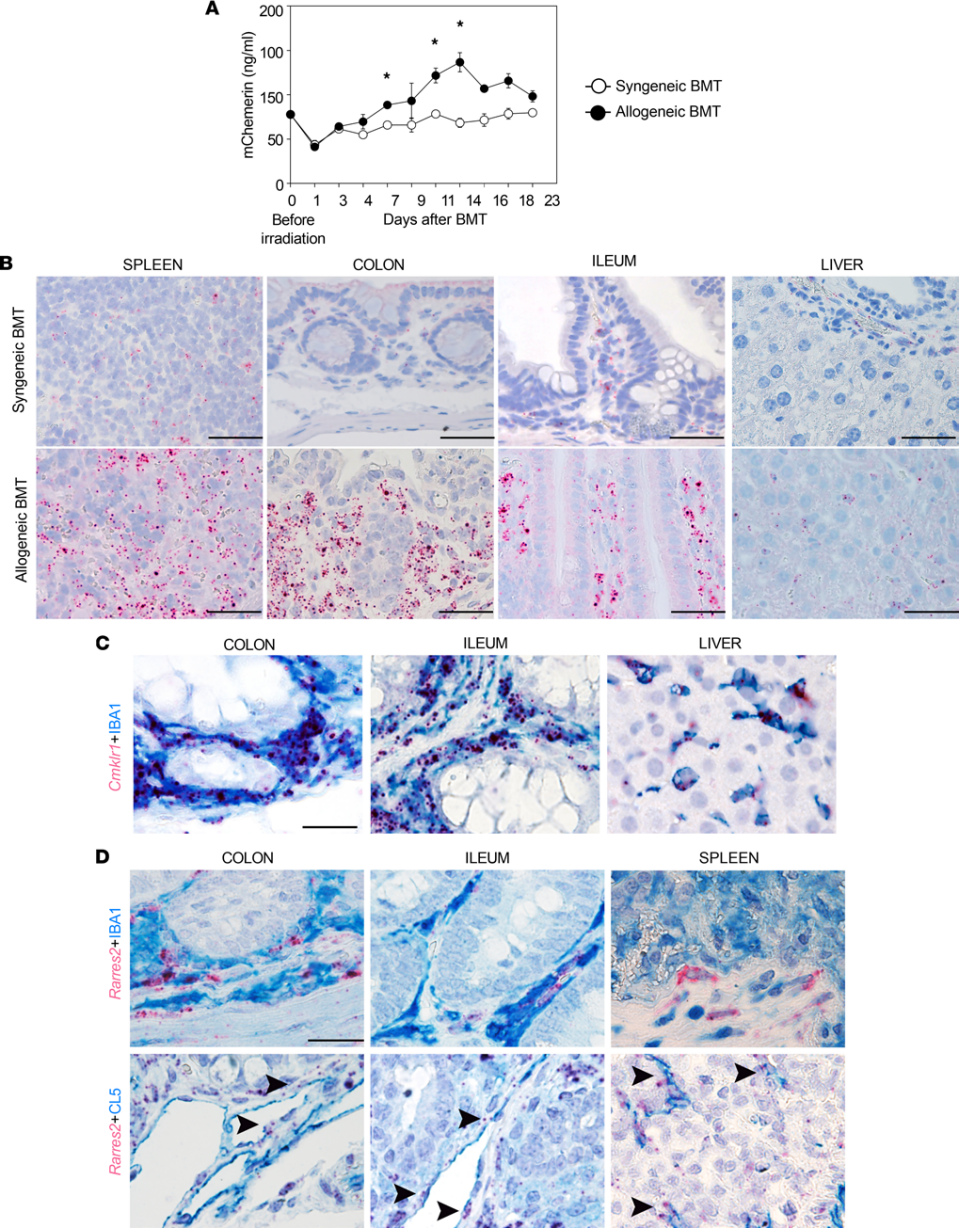
Analysis of chemerin plasma levels and CMKLR1^+^ cells in mice after BMT. Lethally irradiated BALB/c (H-2^d^) mice were transplanted with bone marrow and splenocytes obtained from C57BL/6 WT mice (t-WT, H-2^b^) or BALB/c mice (SYN, H-2^d^). (**A**) Chemerin plasma concentrations were analyzed by ELISA before the conditioning regimen and after BMT at different time points in allogeneic-transplanted mice and syngeneic-transplanted mice. Data are shown as mean ± SEM (*n* ≥ 3), and results are pooled from 2 independent experiments. *Adjusted *P* value < 0.05, Mann-Whitney test with Bonferroni-Dunn correction for multiple comparisons. (**B**) To localize CMKLR1^+^ cells, mouse tissues were analyzed with RNAscope assay. Sections from mouse spleen, colon, ileum, and liver of SYN and allogeneic-transplanted mice were stained for *Cmklr1* mRNA. Original magnification, ×400; scale bars: 50 μm. (**C**) *Cmklr1* transcript (RNAscope, red) was observed in IBA1^+^ macrophages (immunohistochemistry, blue) including liver Kupffer cells. Original magnification, ×600; scale bar: 33 μm. (**D**) The identity of chemerin-producing cells was investigated by RNAscope staining of *Rarres2* mRNA. *Rarres2* staining (red signal) was observed in IBA1^+^ macrophages (immunohistochemistry, blue) and in claudin-5–positive (CL5^+^) endothelial cells (immunohistochemistry, blue; arrowheads, CL5^+^ endothelial cells). Original magnification, ×600; scale bar: 33 μm.

The chemerin/CMKLR1 axis has been implicated in several immune-mediated diseases (11, 17, 21–25). Therefore, the potential role of the chemerin/CMKLR1 axis in the pathogenesis of GvHD was investigated using a major mismatched mouse model of GvHD (B6 into BALB/c mice). Figure 1A shows that in the first 24 hours following total-body irradiation (TBI), chemerin plasma levels displayed an initial transient decrease from baseline (day 0, before irradiation). Subsequently, in allogeneic-transplanted mice, the concentration of chemerin continuously increased, reaching a peak 14 days after bone marrow transplantation (BMT), with levels that were about 75% higher than basal values. On the contrary, in syngeneic-transplanted mice, chemerin levels returned to baseline values and remained stable in the following 3 weeks.

Highly sensitive in situ hybridization assay (RNAscope) was used to evaluate *Cmklr1^+^* cells in different GvHD target organs (Figure 1B). In syngeneic-transplanted mice, *Cmklr1* mRNA was expressed by few scattered cells. On the contrary, a strong reactivity was observed in the spleen, large bowel, small bowel, and liver of allogeneic-transplanted mice compared with syngeneic controls at day +21 after transplantation. Cmklr1 probe did not stain bowel epithelial cells or hepatocytes, whereas it was associated with infiltrating immune cells. *Cmklr1^+^* cells had myeloid-monocytic characteristics and were positive for ionized calcium-binding adapter molecule 1 (IBA1), as highlighted by combined in situ hybridization and immunohistochemistry, demonstrating a macrophage identity (Figure 1C). Furthermore, RNAscope analyses highlighted that *Rarres2* transcript, encoding chemerin, was produced in allogeneic-transplanted mice by a fraction of IBA1^+^ macrophages in colon and ileum, but not by IBA1^+^ splenic macrophages, and by claudin-5–positive (CL5^+^) endothelial cells in colon, ileum, and spleen (Figure 1D). A similar expression was detected in syngeneic-transplanted mice (Supplemental Figure 1; supplemental material available online with this article; https://doi.org/10.1172/jci.insight.154440DS1). Taken together these data indicate that the chemerin/CMKLR1 axis is involved in acute GvHD pathogenesis.

### CMKLR1 exerts a protective role in large-intestinal GvHD.

**Figure 2:**
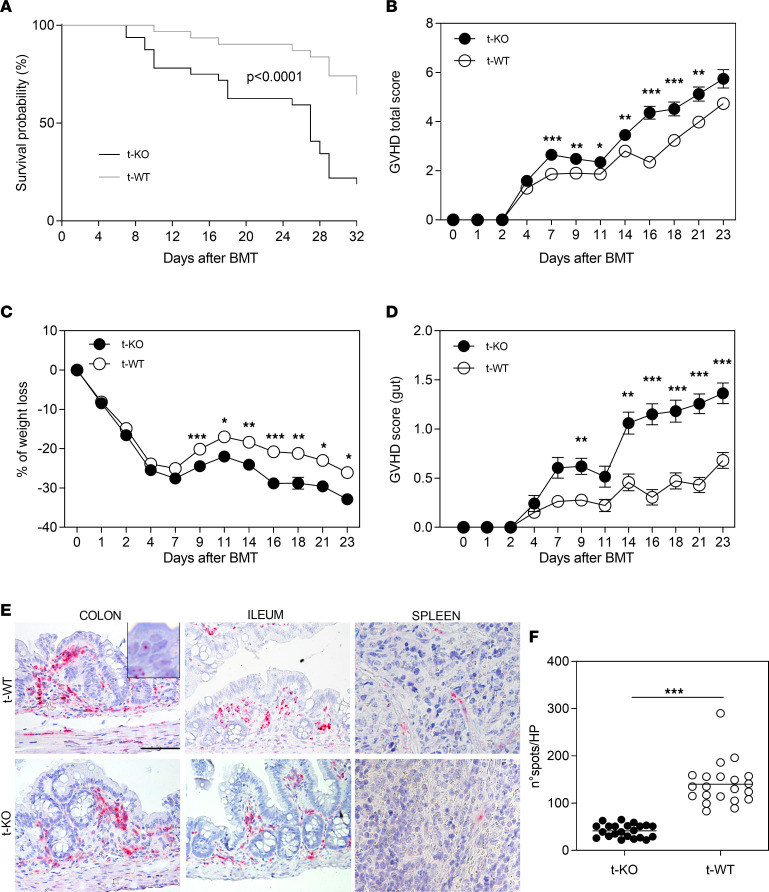
Mice transplanted with CMKLR1-KO donor cells develop severe GI GvHD. Lethally irradiated BALB/c (H-2^d^) mice were transplanted with bone marrow and splenocytes obtained from CMKLR1^–/–^ mice (t-KO, B6, H-2^b^), C57BL/6 WT mice (t-WT, H-2^b^), or BALB/c mice (SYN, H-2^d^). (**A**) The graph shows the survival curves of t-KO mice compared with t-WT mice and syngeneic-transplanted mice. Data are pooled from 2 independent experiments; t-KO (*n* = 32), t-WT (*n* = 31). *P < 0.0001, log-rank (Mantel-Cox) test. (**B**) The graph shows acute GvHD total score. (**C** and **D**) The percentage of weight loss (**C**) and the gut GvHD score (**D**) are illustrated. Data are shown as mean ± SEM. Data are pooled from 2 independent experiments; t-KO (*n* = 33), t-WT (*n* = 36). *Adjusted *P* value < 0.05, **adjusted *P* value < 0.01, ***adjusted *P* value < 0.001, Mann-Whitney test with Bonferroni-Dunn correction for multiple comparisons. (**E**) Mouse colon, ileum, and spleen from t-WT and t-KO were stained with Rarres2 probe. Original magnification, ×400; scale bars: 50 μm. (**F**) *Rarres2^+^* spots were counted in t-KO and t-WT colon sections (*n* = 22 and *n* = 21 fields for t-KO and t-WT mice, respectively). ***P < 0.001, Mann-Whitney test. HP, high-power view.

The role of CMKLR1 in allogeneic BMT was investigated using *Cmklr1-*KO mice as BMT donors. Notably, WT mice transplanted with bone marrow obtained from *Cmklr1*-KO mice (t-KO) developed worse GvHD compared with mice that received an allograft from WT mice (t-WT) (Figure 2). In particular, t-KO mice showed lower survival compared with t-WT mice (Figure 2A, *P* < 0.0001; and Supplemental Figure 2). This result was associated with a higher GvHD clinical score starting from day +7 after BMT (Figure 2B). The increased GvHD score was characterized by increased weight loss (Figure 2C) and diarrhea (Figure 2D) in t-KO compared with t-WT mice. No differences were observed in terms of skin integrity, fur texture, posture, and mouse locomotor activity (data not shown). Interestingly, RNAscope analyses demonstrated that *Rarres2* transcript was upregulated in the colon of t-WT compared with t-KO mice (Figure 2, E and F). These results suggested an organ-specific involvement of the chemerin/CMKLR1 axis in the pathogenesis of intestinal GvHD.

**Figure 3:**
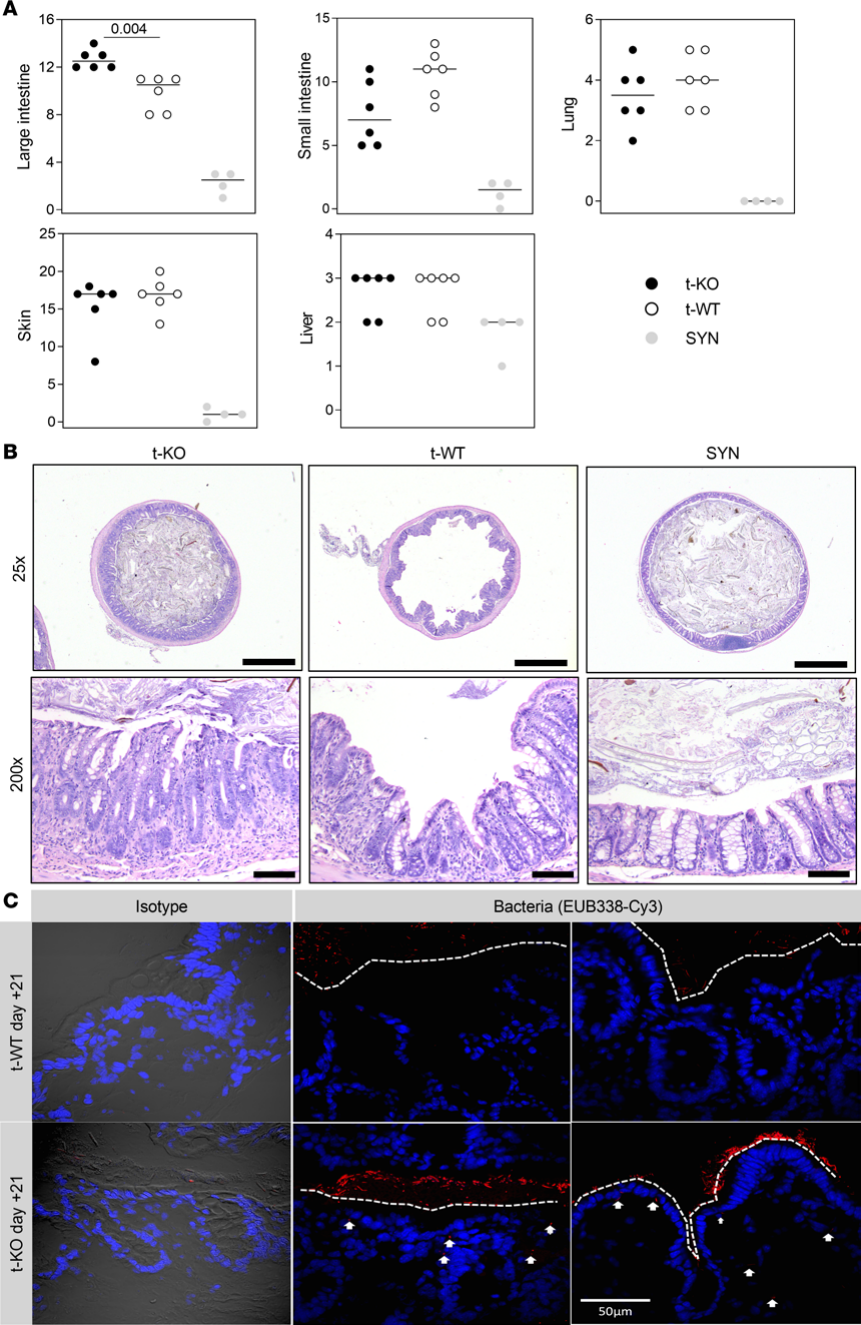
Histopathological analysis of the GvHD target organs large intestine, small intestine, lung, skin, and liver of mice transplanted with CMKLR1-KO donor cells. Lethally irradiated BALB/c (H-2^d^) mice were transplanted with bone marrow and splenocytes obtained from CMKLR1^–/–^ mice (t-KO, B6, H-2^b^), C57BL/6 WT mice (t-WT, H-2^b^), or BALB/c mice (SYN, H-2^d^). Large intestine, small intestine, lung, skin, and liver were harvested 20 days after BMT. H&E staining was used to perform histopathological analysis. (**A**) Large and small intestine total GvHD histological score (range 0–16) and lung, skin, and liver GvHD scores are represented. Data are shown as dot plots with median value (black line); t-KO (*n* = 6), t-WT (*n* = 6), SYN (*n* = 4). *P* values: Mann-Whitney test. (**B**) Representative images of H&E-stained colon from t-KO, t-WT, and SYN mice (×25 scale bars: 1,000 μm; ×200 scale bars: 100 μm). (**C**) Fluorescent microscopy of intestinal microbiota in Carnoy’s-fixed sections of t-WT or t-KO mice. Representative images of colon tissue of t-WT mice (top) and of t-KO (bottom). Bacteria were identified using FISH and universal probe (EUB338-Cy3). Nuclei were visualized using DAPI (blue). Negative control was acquired with Nomarski differential interference contrast. White arrows indicate translocation of bacteria into the mucosa, whereas dashed white lines represent the distance between bacteria and epithelium. The images are representative of 5 different fields per animal and were acquired by confocal microscopy with a 60-oil immersion Olympus Plan Achromat Objective. Scale bar: 50 μm.

Histological analysis performed on GvHD target organs at day +21 after BMT revealed that the colon was the organ mostly affected by GvHD in t-KO mice (Figure 3A). Detailed analysis of the colon at day +21 showed that GvHD difference between t-KO and t-WT mice was mainly attributable to increased colitis and crypt hyperplasia in t-KO mice (Supplemental Figure 3). Conversely, no significant histological differences were found in the number of intraepithelial lymphocytes or in epithelial degeneration (Supplemental Figure 3), nor in the liver, lung, skin, or small intestine, between t-WT and t-KO mice (Figure 3A). In t-KO mice the colon mucosa was more thickened than in t-WT mice, as a result of increased inflammatory cell infiltration and crypt hyperplasia (Figure 3B). In addition, t-KO mice displayed a massive gut bacterial translocation compared with t-WT mice. At day +21 after transplantation, bacteria localized in t-KO intestine very close to the epithelial layer instead of being confined to the mucus layer, distant from the epithelial barrier. This observation supports a defect in the epithelial barrier function in *Cmklr1*–t-KO mice (Figure 3C).

We next evaluated the potential role of C*mklr1* in the development of intestinal GvHD, by using C*mklr1-*KO C57BL/6 mice as BMT recipients of WT donor cells (BALB/c). KO recipient mice showed a significantly decreased survival (*P* < 0.05), associated with a higher number of deaths in the first 2 weeks after transplant (Supplemental Figure 4A). KO mice showed a more severe GvHD (Supplemental Figure 4B), and the gut was once more the organ more involved, as demonstrated by the increased weight loss and GvHD gut score of KO compared with WT mice (Supplemental Figure 4, C and D). These data further support the idea that CMKLR1 exerts a protective role in intestinal GvHD.

**Figure 4:**
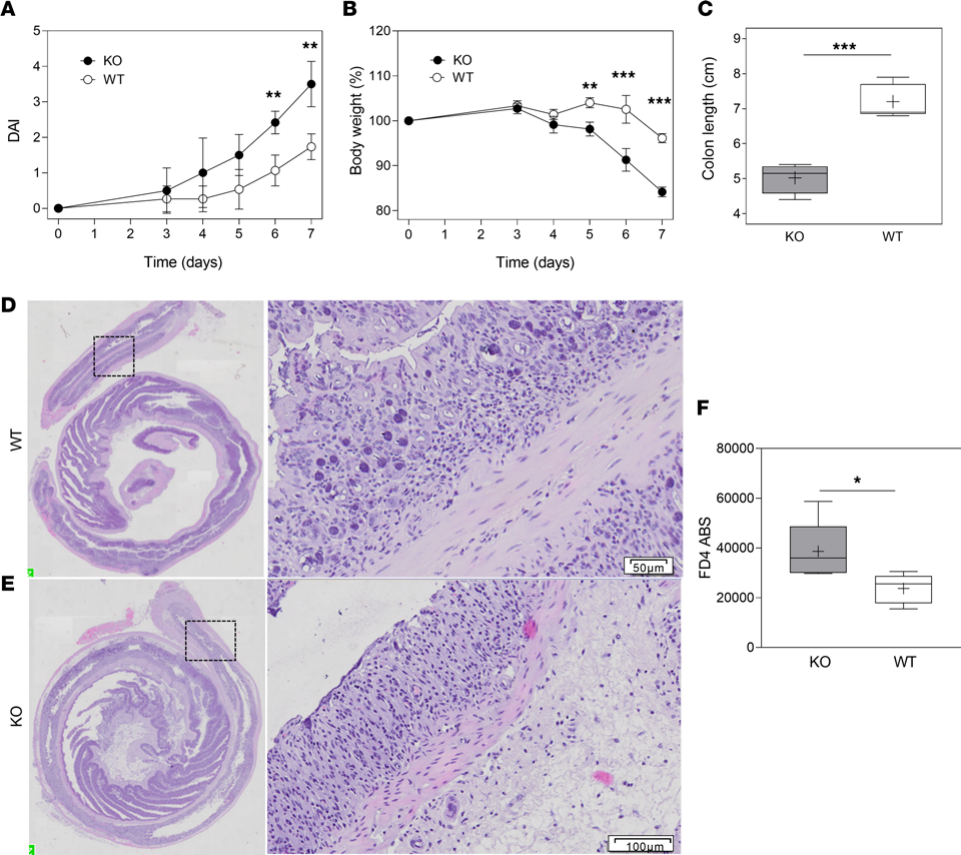
CMKLR1-KO mice show severe intestinal damage in an acute DSS-induced colitis model. Clinical assessment of acute DSS-induced colitis. (**A** and **B**) Mouse disease activity index (DAI) (**A**) was scored by evaluation of bleeding and stool consistency and loss of body weight (**B**) (5 WT mice and 4 CMKLR1-KO mice). Data are represented as mean ± SD. (**C**) Colon length was measured in 5 WT mice and 4 KO mice. Each box plot shows the median (line) and the mean (+) and extends from the lowest to the highest value. **Adjusted *P* value < 0.01, ***adjusted *P* value < 0.001, unpaired *t* test with Bonferroni-Dunn correction for multiple comparisons (**A** and **B**) or unpaired 2-tailed *t* test (**C**). (**D** and **E**) Representative images of H&E-stained colon sections from WT and CMKLR1-deficient mice. Inset scale bar: 50 μm (**D**) or 100 μm (**E**). (**F**) FITC-dextran (FD4) transepithelial permeability assay in KO versus WT mice treated with DSS to induce colitis. Each box plot shows the median (line) and the mean (+) and extends from the lowest to the highest value. *P < 0.05, Mann-Whitney test. ABS, absorbance.

In order to address the role of CMKLR1 in a more specific mouse model of intestinal inflammation, we evaluated the susceptibility of *Cmklr1*-KO mice in a model of acute dextran sulfate sodium–induced (DSS-induced) colitis. Starting from day +5 after DSS administration, both WT and *Cmklr1*-KO mice displayed an increase in clinical colitis (Figure 4A). Furthermore, compared with WT littermate mice, *Cmklr1*-KO mice were characterized by a significantly higher loss in body weight and a progressive increase in the overall disease activity index (Figure 4, A and B). At sacrifice, *Cmklr1*-KO mice showed shorter colon length, an additional parameter for the evaluation of experimental colitis (Figure 4C). Histological analysis of the mucosa verified more severe microscopic inflammation, epithelial disruption, and loss of crypt architecture in *Cmklr1*-KO colon tissues versus WT ones (Figure 4, D and E), supporting the involvement of CMKLR1-expressing cells in the protection from colitis development. The compromising of the epithelial barrier in *Cmklr1*-KO mice was further confirmed by a FITC-dextran (FD4) transepithelial permeability assay, which demonstrated an increased presence of the fluorescent compound in the serum of KO compared with WT mice (Figure 4F).

### Large intestine of t-KO mice is characterized by a more severe proinflammatory infiltrate.

**Figure 5:**
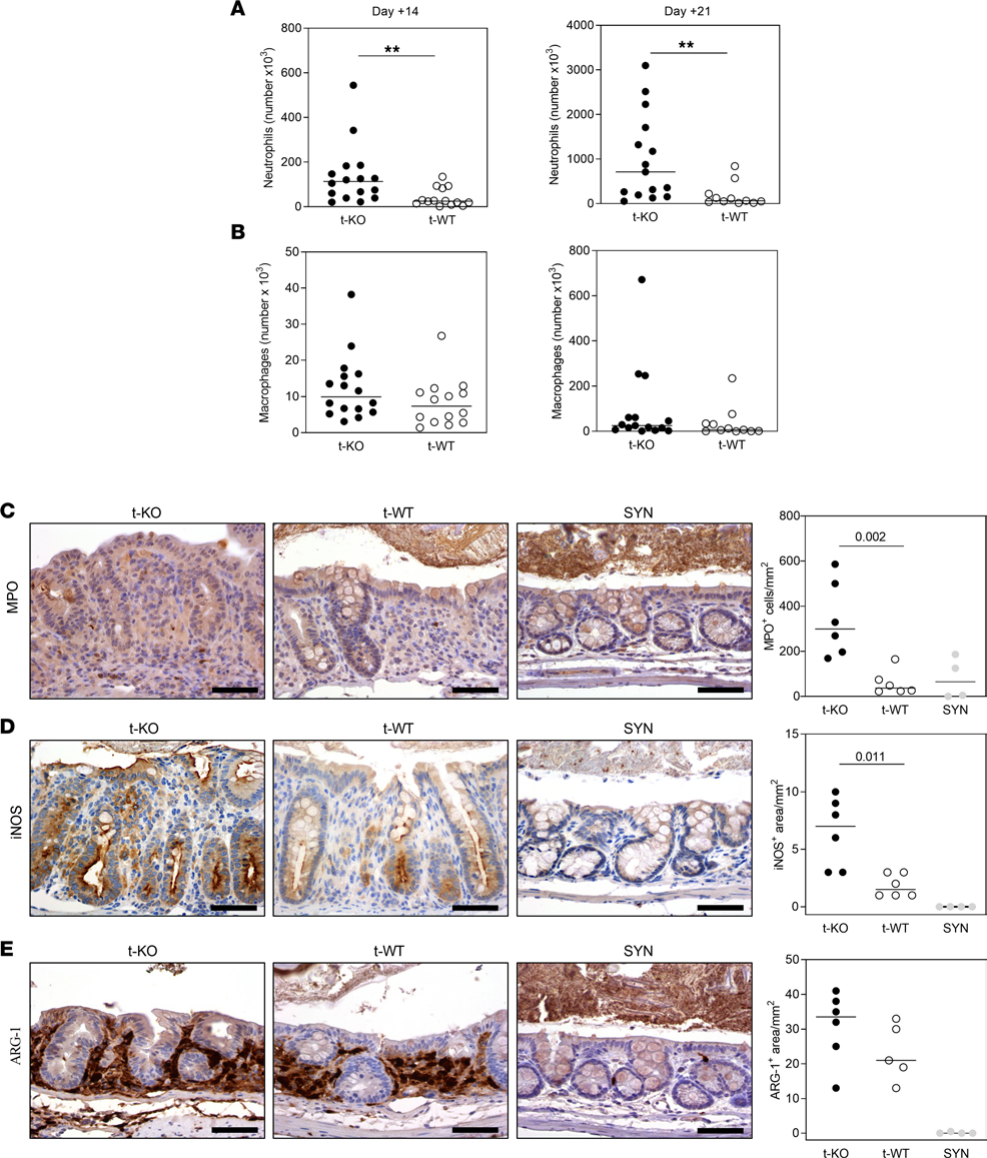
Characterization of the immune infiltrate in the large intestine of mice transplanted with CMKLR1-KO donor cells. Lethally irradiated BALB/c (H-2^d^) mice were transplanted with bone marrow and splenocytes obtained from C57BL/6 CMKLR1*^–/–^* mice (t-KO, B6, H-2^b^) or C57BL/6 WT mice (t-WT, H-2^b^). Large intestine was harvested +14 and +21 days after BMT. Flow cytometry analyses were performed to evaluate immune cell infiltration in colon mucosa. (**A**) Neutrophils were evaluated as percentage of CD11b^+^Ly6C^int^Ly6G^+^ cells on gated CD45^+^ cells. (**B**) Macrophages were evaluated as percentage of CD11b^+^Ly6C^–^F4/80^+^ cells on gated CD45^+^ cells. Their absolute numbers are represented as dot plots; the black bar indicates the median. Data are pooled from 2 independent experiments; t-KO (*n* = 16), t-WT (*n* = 14) at day +14; t-KO (*n* = 15), t-WT (*n* = 11) at day +21. *P* values: Mann-Whitney test. **P value < 0.01. (**C**–**E**) Large intestine was harvested 20 days after BMT, and immunoperoxidase staining was performed to detect the expression of MPO, iNOS, and ARG-1 into the tissue. Representative images of MPO (**C**), iNOS (**D**), and (**E**) ARG-1 staining in t-KO, t-WT, and SYN and their quantification as number of positive cells/mm^2^ or immunoreactive area/mm^2^ of colon mucosa are shown (×400 scale bars: 50 μm). Data are represented as dot plots; the black bar indicates the median. t-KO (*n* = 6), t-WT (*n* = 6), SYN (*n* = 4). *P* values: Mann-Whitney test.

The inflammatory infiltrate was characterized in t-KO BM-transplanted mice. Flow cytometry analyses (gating strategy described in Supplemental Figure 5) showed that the total number of macrophages, within CD45^+^ cells (Supplemental Figure 6), was similar between t-KO and t-WT mice (Figure 5B). Furthermore, we investigated by RNAscope the expression of *Cmklr1* in IBA1^+^ macrophages resident in the gut. As reported in Supplemental Figure 7, double-positive cells were detected in both t-WT and t-KO mice. On the contrary, total neutrophil count in the large intestine of t-KO was increased at days +14 and +21 after BMT, in comparison with t-WT mice (Figure 5A and Supplemental Figure 6), supporting a role for these cells in the severity of GvHD in the large intestine. Immunohistochemistry also revealed that, 21 days after BMT, the colon of t-KO mice was infiltrated by a higher number of cells positive for myeloperoxidase (MPO) and inducible NO synthase (iNOS) than that of t-WT animals (Figure 5, C and D). The increase of MPO and iNOS markers in t-KO mice was also confirmed at an earlier disease stage (day +14 after BMT; Supplemental Figure 8), suggesting that the increase of neutrophil infiltrate in t-KO mice is a precocious event, possibly responsible for the severe gut damage observed in t-KO mice. On the contrary, the expression of the antiinflammatory marker arginase-1 (ARG-1) was similar in the 2 experimental conditions at all the time points analyzed (Figure 5E and Supplemental Figure 8). Furthermore, the number of total T and B cells in the colon of t-KO and t-WT mice was similar, as evaluated both by immunohistochemistry and flow cytometry at different time points (Supplemental Figures 8 and 9).

**Figure 6:**
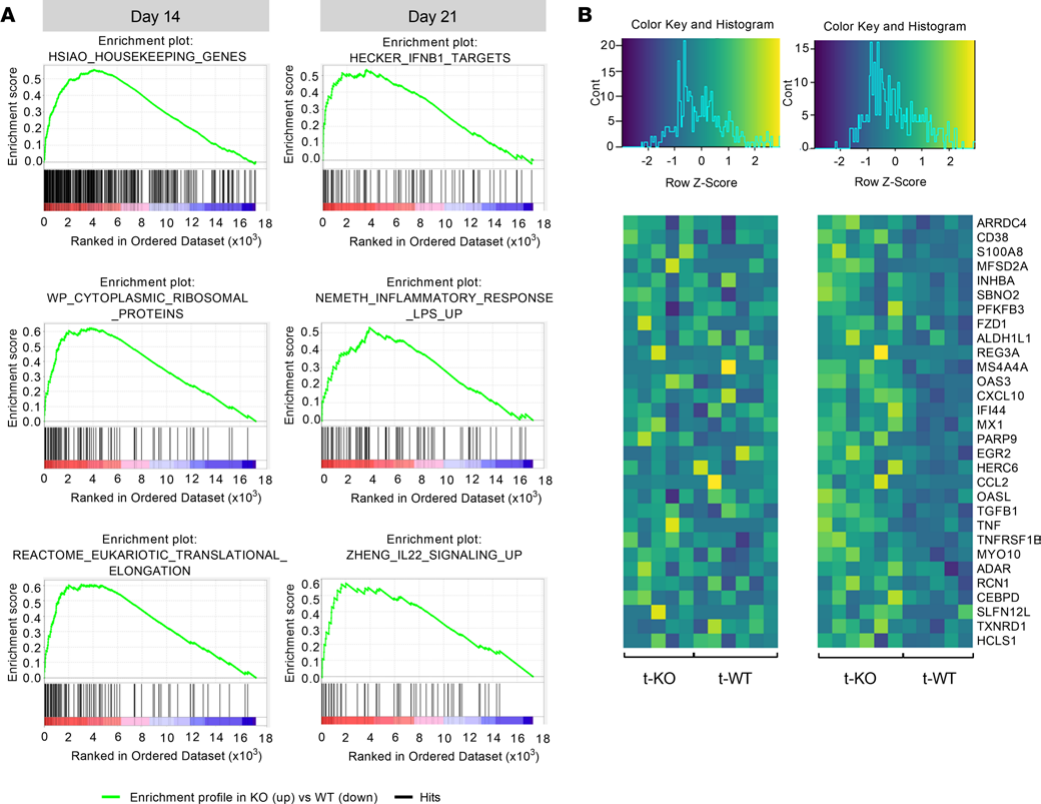
Whole-transcriptome analysis by next-generation sequencing of the large intestine of mice transplanted with CMKLR1-KO or -WT donor cells. Lethally irradiated BALB/c (H-2^d^) mice were transplanted with bone marrow and splenocytes obtained from C57BL/6 CMKLR1*^–/–^* mice (t-KO, B6, H-2^b^) or C57BL/6 WT mice (t-WT, H-2^b^). RNA sequencing experiments by next-generation sequencing were performed on paraffin-embedded large intestine sections harvested 14 and 21 days after BMT from t-KO and t-WT mice (*n* > 5 for each group at each time point). (**A**) Top enriched gene sets identified at days 14 (left) and 21 (right) by GSEA of transcripts. (**B**) Heatmap representation of the top 10 ranked genes of each day 21 gene set represented in **A**.

To further analyze at the molecular level the inflammatory pathways involved in GvHD pathogenesis, we performed RNA-sequencing experiments (whole-transcriptome analysis by next-generation sequencing) on paraffin-embedded colon sections obtained at day +14 and +21 after BMT from t-KO and t-WT mice. At day +14, differentially expressed genes (DEGs) were prevalently related to the activation of transcription/translation processes; on the contrary, DEGs at day +21 were mainly related to inflammatory and tissue regeneration processes (Figure 6A). Accordingly, gene set enrichment analysis (GSEA) of transcripts identified IFNB1 targets, inflammatory response against bacterial lipopolysaccharide, and IL-22 signaling as top enriched gene sets, suggesting once more an upregulation of inflammation-related and tissue regeneration pathways in t-KO compared with t-WT mice (Figure 6A). The heatmap representation of the top 10 ranked genes for each above-mentioned gene set showed a random distribution between t-KO and t-WT mice at day +14, but clearly highlighted a differential expression pattern at day +21 between the 2 groups (Figure 6B). In detail, at day +21, several inflammatory genes appeared consistently upregulated in t-KO mice, including those encoding TNF, IFN-γ–inducible protein 10 (IP-10; CXCL10), and CD38, which controls inflammatory processes in the colon (31). Another gene upregulated in t-KO mice encodes the S100A8 protein, a damage-associated molecular pattern molecule abundant in the cytoplasm of neutrophils and released, upon activation, through the formation of neutrophil extracellular traps (32). Notably, S100 proteins were found elevated in the stool and in the serum of patients with acute GvHD and in inflamed bowel tissue from patients with acute intestinal GvHD (33). Furthermore, 2 genes encoding TGF-β family proteins and induced under inflammatory conditions in the attempt to control inflammation, namely *Inhba* and *Tgfb1*, were overexpressed in t-KO mice. Interestingly, *Inhba* encodes the subunits of activin A, a protein with a prominent role in the control of bacterial infections, which is upregulated in critically ill patients with sepsis and predicts disease severity (34). TGF-β1, instead, is a protein with a well-recognized role in the control of intestinal inflammation and has been reported to be elevated in the serum of patients with ulcerative colitis (35). In addition, several genes related to IL-22 signaling and involved in tissue protection from damage and regeneration were identified as enriched in t-KO mice by GSEA. One of them is regenerating family member 3α (*Reg3a*), which encodes an acute-phase lectin that in its mature form functions as an antimicrobial protein and promotes intestinal stem cell survival. *Reg3a* transcript enrichment in t-KO mice suggests it could be induced by the increased colon damage and bacterial translocation observed in this experimental group in the attempt to induce tissue regeneration. Interestingly, REG3α plasma levels have been demonstrated to correlate with disease activity in inflammatory bowel disease and in intestinal GvHD (1).

Taken together, the data indicate an exacerbated inflammatory process in t-KO mice. A tendency of increased activation of some antiinflammatory markers may represent the ultimate effort to contain the inflammatory process.

### Adoptive transfer of WT monocytes ameliorates colon GvHD damage.

**Figure 7:**
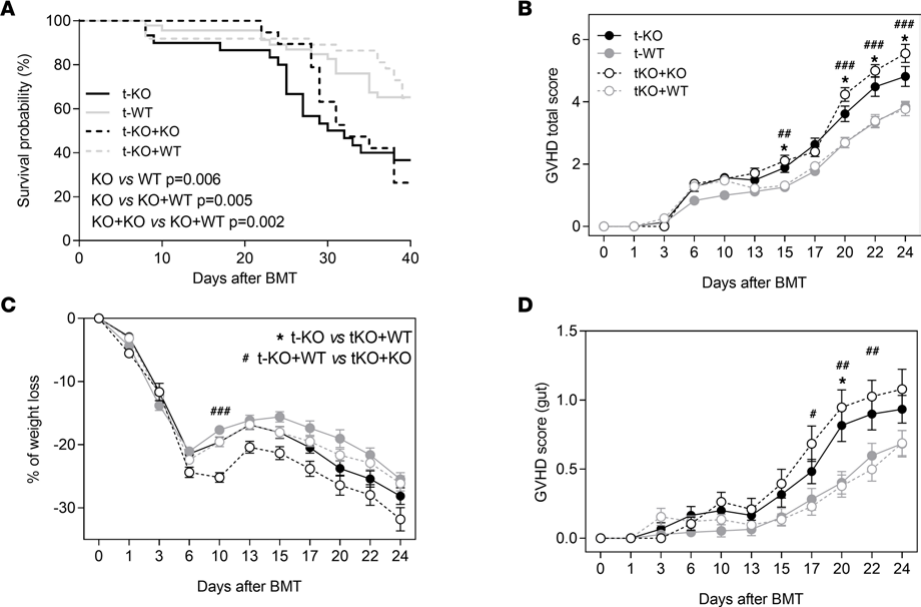
Adoptive transfer of CMKLR1-expressing monocytes in KO-transplanted mice improves GI GvHD. Lethally irradiated BALB/c (H-2^d^) mice were transplanted with bone marrow and splenocytes obtained from CMKLR1*^–/–^* mice (t-KO, B6, H-2^b^) or C57BL/6 WT mice (t-WT, H-2^b^). CMKLR1*^–/–^* cells were also transplanted in combination with purified CMKLR1*^–/–^* monocytes (t-KO+KO) or purified WT monocytes (t-KO+WT). (**A**) Survival curves of t-KO–transplanted mice with added WT or CMKLR1*^–/–^* monocytes. Data are pooled from 5 independent experiments; t-KO (*n* = 30), t-WT (*n* = 46), t-KO+KO monocytes (*n* = 19), t-KO+WT monocytes (*n* = 37). *P* values: log-rank (Mantel-Cox) test. (**B**) GvHD total score was assigned as previously described. GvHD total score confirmed that the adoptive transfer of WT monocytes improved GvHD. (**C**) Mice were weighed every 2–4 days in order to evaluate the involvement of the GI tract. (**D**) Mice were evaluated for the presence of diarrhea, as described above. Data are shown as mean ± SEM. Data are pooled from 5 independent experiments; t-KO (*n* = 30), t-WT (*n* = 46), t-KO+KO monocytes (*n* = 19), t-KO+WT monocytes (*n* = 37). *Adjusted *P* value < 0.05, t-KO vs. t-KO+WT monocytes; ^#^adjusted *P* value < 0.05, ^##^adjusted *P* value < 0.01, ^###^adjusted *P* value < 0.001, t-KO+WT monocytes vs. t-KO+KO monocytes; Mann-Whitney test with Bonferroni-Dunn correction for multiple comparisons.

Monocytes and their progeny play an important role in modulating inflammation. In addition, they express CMKLR1 (36, 37). Indeed, we evaluated whether they could play a role in controlling exacerbated gut inflammation of t-KO mice. This hypothesis was tested by the adoptive transfer of WT monocytes into t-KO mice. Purified monocytes from *Cmklr1*-KO and WT mice were inoculated in randomized t-WT and t-KO mice. As expected, t-KO mice developed more severe GvHD compared with t-WT mice, and the administration of monocytes from *Cmklr1*-KO mice (t-KO+KO monocytes) did not change the phenotype in terms of either survival or GvHD clinical score. On the contrary, the adoptive transfer of WT monocytes to t-KO mice (t-KO+WT monocytes) significantly improved t-KO mouse survival (*P* < 0.005, t-KO+WT monocytes vs. t-KO+KO monocytes) (Figure 7A). Similarly to t-WT mice, t-KO+WT monocytes showed reduced total GvHD clinical score (Figure 7B), which corresponds to a decreased weight loss score (Figure 7C) and significantly lower GI GvHD (Figure 7D). Another CMKLR1-expressing immunosuppressive cell population, namely plasmacytoid dendritic cells (pDCs), were described to dampen GvHD by inducing the generation of regulatory T cells (Tregs) (38). Our data indicate that the adoptive transfer of WT pDCs to t-KO mice did not significantly impact on GvHD course. Indeed, t-KO mice that received WT pDCs were comparable to t-KO mice in terms of both survival and intestinal GvHD, as demonstrated by weight loss monitoring and GvHD gut score (Supplemental Figure 10).

**Figure 8:**
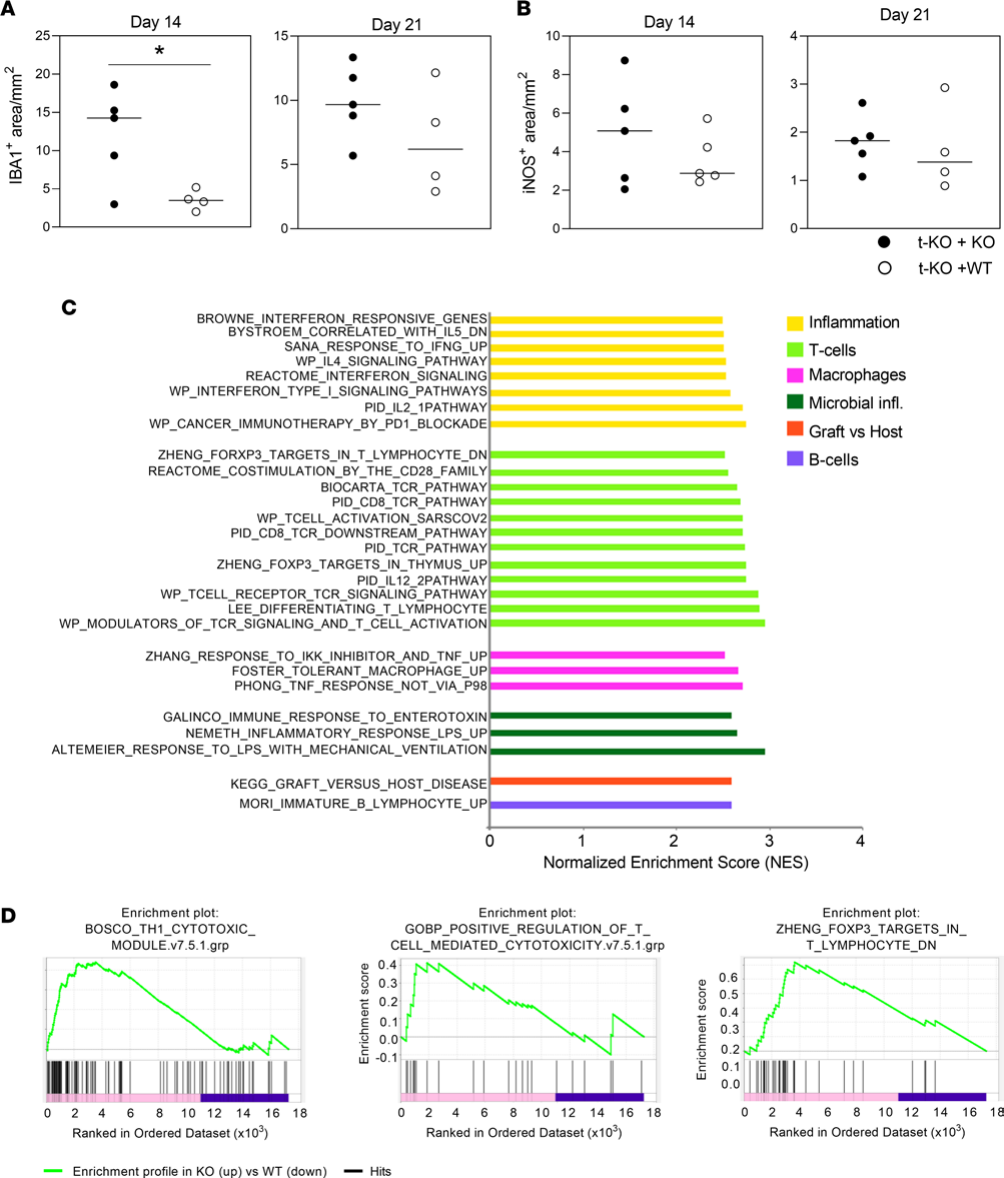
Characterization of the mechanism of action used by adoptively transferred CMKLR1-expressing monocytes to mitigate intestinal GvHD in mice transplanted with CMKLR1-KO donor cells. Large intestine was harvested from lethally irradiated BALB/c (H-2^d^) mice at +14 and +21 days after transplantation of bone marrow and splenocytes obtained from CMKLR1^–/–^ mice (B6, H-2^b^) in combination with purified CMKLR1^–/–^ monocytes (t-KO+KO) or purified WT monocytes (t-KO+WT). (**A** and **B**) Immunoperoxidase staining was performed on paraffin-embedded sections of large intestine to detect the expression of IBA1 (**A**) and iNOS (**B**) into the tissue. Marker quantification as immunoreactive area/mm^2^ of colon mucosa is shown. Data are represented as dot plots; the black bar indicates the median. t-KO+KO (*n* = 5, at both time points), t-KO+WT (*n* ≥ 4, at both time points). *P < 0.05, unpaired 2-tailed *t* test. (**C**) Whole-transcriptome analysis was performed by next-generation sequencing on OCT-embedded snap-frozen colon tissues collected from t-KO+KO and t-KO+WT mice (day +14 and day +21, *n* = 5 and 10 for each group, respectively, distributed in the 2 time points). GSEA was performed on day 21 transcripts. The bar graph represents relevant gene pathways upregulated in colons from t-KO+KO monocytes. (**D**) Enrichment plots representing the running enrichment score of 3 selected gene sets (day 21).

To better understand whether the adoptive transfer of CMKLR1-expressing WT monocytes could impact on the local inflammatory microenvironment in the gut, we firstly performed immunohistochemistry on OCT-embedded snap-frozen colon sections. As shown in Figure 8A, the number of cells positive for the myeloid activation marker IBA1 was clearly decreased in t-KO+WT monocyte–transplanted compared with t-KO+KO monocyte–transplanted mice. A similar trend was observed for iNOS^+^ cells (Figure 8B), with the effect being most evident for both markers at day +14. The antiinflammatory action of adoptively transferred monocytes was further documented by RNA sequencing (whole-transcriptome analysis by next-generation sequencing) of OCT-embedded snap-frozen colon tissues. Data depicted in Figure 8, C and D, highlighted that multiple gene pathways related to immune system activation and inflammation were upregulated in colons from t-KO+KO monocyte–transplanted mice on day +21. Specifically, the colon of mice receiving only CMKLR1-KO donor cells showed a more prominent activation of T cell response, TCR signaling, and IL-2/IL-2 receptor pathways, crucially involved in T cell proliferation. Notably, the analysis of the T cell compartment suggests that the adoptive transfer of WT monocytes decreased the cytotoxic potential of effector T cells, and shifted the Th1/Treg balance in favor of Tregs (Figure 8D). Transcripts related to B cell receptor signaling and dendritic cell maturation were also enhanced in the t-KO+KO monocyte group, suggesting a general exacerbated activation of tissue-resident immune cells (Figure 8C). Importantly, inflammatory pathways (e.g., IL-12, type I IFNs, TNF, IL-6, metalloproteases, etc.) and antimicrobial immune responses (activation pathways downstream of bacterial lipopolysaccharide, immune response to enterotoxins, defensins, antimicrobial peptides, etc.) were increased in t-KO+KO–derived monocytes compared with t-KO+WT–derived cells, possibly as a result of massive microbial translocation caused by GvHD in damaged tissue. Along with increased inflammation, transcripts annotated into the Kyoto Encyclopedia of Genes and Genomes (KEGG) pathway “Graft versus Host Disease” were enriched in the t-KO+KO monocyte group, as a consequence of the more severe intestinal disease (Figure 8C).

Overall, the new data support the hypothesis that infusion of WT monocytes can mitigate GvHD manifestations by decreasing gut inflammation and activation of infiltrating immune cells.

### Chemerin plasma levels in a cohort of hematopoietic stem cell transplantation patients.

**Figure 9:**
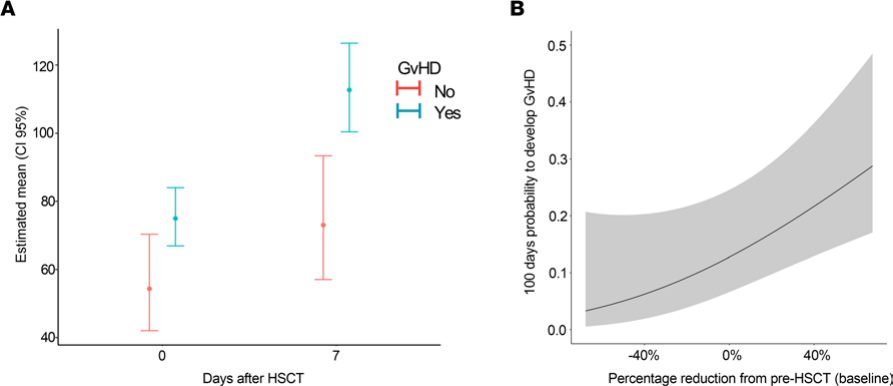
Analysis of chemerin plasma levels in a cohort of HSCT patients. Chemerin plasma levels were evaluated by ELISA in the peripheral blood of 71 pediatric HSCT patients. Among enrolled patients, 59 developed GvHD within 100 days from HSCT, while 12 did not. (**A**) Estimated marginal means and corresponding 95% CIs for chemerin levels at different time points (left: week 0 = after conditioning, before HSCT; right: 1 week after HSCT). Estimates are adjusted for baseline levels (set to global average). (**B**) Estimated probability of developing GvHD within 100 days and corresponding 95% confidence bands as a function of percentage variation of chemerin levels at week 0 versus baseline values [estimated as (baseline – week 0)/baseline].

Based on the results obtained in the mouse model, chemerin circulating levels were evaluated in a cohort of hematopoietic stem cell transplantation (HSCT) pediatric hemato-oncological patients at risk of acute GvHD. Plasma samples were collected from 71 pediatric patients before conditioning regimen (baseline), on day 0 (HSCT), and weekly up to day +100 after HSCT. Among enrolled patients, 59 developed GvHD, while 12 did not. Patients’ characteristics, including underlying disease, HSCT type, and GvHD prophylaxis, incidence, and severity are summarized in Tables 1, 2, and 3. Chemerin plasma levels slightly decreased at day 0 (geometric mean 70.03 ng/mL ± 1.68 SD) compared with baseline (geometric mean 92.43 ng/mL ± 1.64 SD) with a 24.2% reduction (CI_95%_ 13.8%–33.3%, *P* < 0.001), independent of conditioning regimen (*P* = 0.26, data not shown). Before conditioning treatments, chemerin concentration was similar in all the patients independent of whether they developed GvHD (fold change 0.95, CI_95%_ 0.70–1.27, *P* = 0.71). However, variation of chemerin levels on days 0 and +7 between the 2 groups was significantly different, and this difference was even more evident after normalization on baseline levels. Figure 9A shows that chemerin levels on days 0 and +7 after HSCT (adjusted for preconditioning baseline values) were higher in patients who developed GvHD compared with the no-GvHD group (75.0 vs. 54.4 ng/mL, fold change = 1.38, CI_95%_ 1.04–1.83, *P* = 0.026). Similarly, on day +7 chemerin levels were 1.54-fold higher in patients with GvHD compared with no-GvHD (112.7 vs. 73.9 ng/mL, fold change 1.54, CI_95%_ 1.18–2.03, *P* = 0.02). Moreover, the percentage variation of chemerin levels between day 0 and baseline values was predictive of GvHD development during the 100 days after HSCT (Figure 9B). Higher probability of developing GvHD was associated with a higher increase of chemerin levels with respect to baseline levels (HR 0.93 for a 10% increase in chemerin, CI_95%_ 0.88–0.98, *P* = 0.006).

Overall, the strong correlation observed between chemerin levels in the first week after HSCT and the onset of acute GvHD suggests the involvement of this protein in disease pathogenesis.

## Discussion

GI GvHD is one of the most frequent causes of death after allogeneic BMT (2) because of the related diagnostic and therapeutic challenges (39). So far, the identification of new highly targeted treatments represents an urgent clinical need for those patients who, not responding to first-line corticosteroids, have a very poor outcome (39). T cells are the main effector cells involved in GvHD pathogenesis; innate immune cells, such as myeloid cells with antigen-presenting and cytokine-producing functions, also play crucial roles (40–46). Chemerin is a chemotactic factor that through interaction with the CMKLR1 receptor can induce the migration of APCs, such as conventional and pDCs (14, 15) and macrophages (20), as well as of natural killer cells (21, 47). Two other GPCRs, namely CCRL2 and GPR1, can bind chemerin, but their ability to transduce intracellular signals and promote cell activation is still unclear (17). In the last decade, several studies have focused on the role of the chemerin/CMKLR1 axis in immune-mediated diseases (21, 22, 24, 26, 27). For instance, circulating levels of chemerin strongly correlated with markers of inflammation, such as TNF-α, IL-6, and C-reactive protein, in the context of obesity (48, 49). Elevated chemerin plasma levels were also found associated with inflammatory intestinal diseases (50), such as ulcerative colitis (51, 52), chronic pancreatitis (53), hepatitis (54), and tumors (28, 29).

This study was performed to investigate the role of chemerin in the pathogenesis of acute GvHD using a major mismatched GvHD mouse model that recapitulates the pathology associated with allogeneic HSCT. Here we report that after total-body irradiation, chemerin plasma levels underwent a transient decrease followed by a marked increase that was specifically observed in allogeneic-transplanted mice developing GvHD. The possible involvement of the chemerin/CMKLR1 axis in GvHD was further investigated in *Cmklr1*-KO mice. Clinical and histopathology scoring showed that mice transplanted with *Cmklr1^–/–^* cells (t-KO) developed a more severe GI GvHD, associated with colitis, as compared with WT-transplanted (t-WT) mice. An exacerbated inflammatory phenotype and an increased intestinal permeability were also observed in *Cmklr1-*KO mice in the model of DSS-induced colitis. Previously, it was reported that *Cmklr1*-KO mice were more susceptible in a model of acute lipopolysaccharide-induced lung inflammation (13) and viral pneumonia (55). In both conditions, *Cmklr1*-KO mice showed increased infiltration of the lungs by neutrophils, macrophages, and dendritic cells. Similarly, *Cmklr1*-KO mice used both as transplant recipients of our GvHD model and in a DSS-induced colitis model showed a very severe intestinal pathology, associated with an increased tissue inflammation. In this regard, Lin and colleagues demonstrated that deficiency of chemerin or intestinal epithelial cell–specific CMKLR1 conferred high susceptibility to microbiota-driven neutrophilic colon inflammation in mice following epithelial injury (56). Concerning the GvHD model, a high mortality rate was observed in allogeneic-transplanted *Cmklr1*-KO recipient mice in the early days after irradiation, suggesting an increased susceptibility to the conditioning regimen. The increased intestinal manifestations later on presented by *Cmklr1*-KO recipient mice further point to increased tissue damage, possibly mediated by GvHD effectors. Future studies will be necessary to clarify the molecular mechanisms underlying the increased GvHD severity in *Cmklr1*-KO recipient mice.

GvHD in t-KO mice was characterized by an extensive inflammatory infiltrate in the large intestine with a marked expression of MPO, a neutrophilic marker, and of iNOS; no difference was observed in lymphoid cell markers between t-KO and t-WT mice. Flow cytometry verified the increased presence of infiltrating neutrophils in t-KO mice, suggesting an effector role for these cells in severe GvHD damage. Since neutrophils do not express CMKLR1 (17), their recruitment can be considered an epiphenomenon of severe intestinal damage caused by the absence of CMKLR1 expression in other leukocyte subsets. Neutrophils are known to be crucial in mediating intestinal GvHD (5, 57). Neutrophils are recruited to the small intestine lamina propria after radiation injury and promote inflammation through the release of proinflammatory molecules, such as reactive oxygen species (ROS) and MPO, and through antigen presentation to T cells in mesenteric lymph nodes (58). Neutrophil accumulation during GvHD pathogenesis was associated with bacterial leakage into peri-intestinal tissues (5). Accordingly, the increased infiltration of neutrophils observed in t-KO animals was associated with massive bacteria translocation, as shown by FISH analysis. Conversely, T and B cell compartments did not appear to be particularly involved in our experimental conditions. The analyses of colon transcriptome confirmed, at the molecular level, an increased inflammatory state in t-KO mice, along with increased tissue damage and induction of antimicrobial pathways possibly triggered by translocated bacteria. Notably, chemerin production was decreased in the colon of t-KO mice. This finding is reminiscent of the reduction in chemerin production previously described in the inflamed epidermis of psoriatic plaques, suggesting that at the epithelial barriers, chemerin may be part of the homeostatic program (24). In agreement with this observation, chemerin reduction was correlated with increased susceptibility to inflammation of the colon (56).

The role of macrophages in the pathophysiology of acute and chronic GvHD has been recently highlighted (7), with a direct role of donor monocyte-derived macrophages in promoting human GvHD being demonstrated in cutaneous GvHD lesions (6). However, macrophages present a high degree of heterogeneity and plasticity in relation to the microenvironmental signals (59, 60), and the protective role of macrophages in different models of experimental colitis was also documented (61, 62). In this study we report that the adoptive transfer of CMKLR1-competent monocytes to t-KO mice was able to mitigate GvHD pathology and severity. Molecular analyses of gut transcriptome demonstrated that the protective action of donor-derived WT monocytes was achieved through a general dampening of the tissue inflammatory response associated with the modulation of several immune cell subsets. Some of the most influenced pathways that could account for GvHD modulation included inhibition of cytotoxic Th1 response, accompanied by induction of Tregs, downregulation of dendritic cell maturation, and B cell activation. Furthermore, the downmodulation of pathways related to antimicrobial responses in t-KO mice that received WT monocytes suggests, also at the molecular level, an amelioration of the gut barrier function. Overall, these data clearly indicate that functional CMKLR1/chemerin axis is required for the control of GvHD-related gut inflammation. In line with these results, chemerin administration was found to exert an antiinflammatory action associated with the increased infiltration of macrophages in a model of lung inflammation (13), and depletion of intestinal mononuclear phagocytes in a DSS-induced colitis model was shown to increase colitis severity owing to a higher infiltration of MPO^+^ neutrophils (62). A recent report also showed the involvement of formyl peptide receptor 2 in the recruitment of monocytes to the intestine during the resolution of inflammation and wound repair (63). Furthermore, in the context of colon rectal cancer, it has been recently demonstrated that chemerin may promote, in concert with other cytokines such as TGF-β and IL-1, the interaction between SPP1^+^ macrophages and fibroblasts (64). This interaction was crucial to promote the formation of immune-excluded tumor desmoplastic structures able to limit T cell infiltration (64). Taken together, these results suggest that CMKLR1 may be an important signal required for the regulatory effect of mono-derived macrophages on intestinal GvHD inflammation and injury. Although in an indirect manner, the finding that IBA1^+^ *Cmklr1^+^* macrophages are similarly detected in t-WT and t-KO large intestine suggests that CMKLR1^+^ cells are likely to play their role in the control of GvHD in tissues other than intestine. In this regard, it is interesting to note that CMKLR1 was suggested to act as a lymph node homing molecule (15). Further studies will be needed to dissect whether the GvHD-modulating action of CMKLR1^+^ monocytes and their progeny is achieved thanks to their direct migration to the gut, in response to locally produced chemerin, or to lymphoid organs, where they could modulate antigen presentation and activation of adaptive immunity.

Interestingly, the finding that WT pDCs, a cell subset endowed with a CCR9-dependent gut homing potential (38) and previously shown by our group to express CMKLR1/ChemR23 (14), were not able to improve the disease course leaves monocytes as the only CMKLR1^+^ cell population endowed with gut antiinflammatory functions. Our data are in contrast to a previous report in which adoptive transfer of CCR9^+^ pDCs sorted from Flt3L-treated mice was shown to control GvHD (38). The reasons for this difference might be, at least in part, explained by the different GvHD models used (T cell–depleted versus –undepleted) and the different source of adoptively transferred pDCs (in vivo–expanded/CCR9^+^-sorted pDCs versus total unmanipulated spleen-derived pDCs).

Our preliminary data, obtained in a cohort of 71 HSCT patients, also show that the increase of chemerin plasma levels during the first week following HSCT can markedly predict the development of acute GvHD, with lower chemerin levels being protective. Similarly, in our GvHD mouse model, chemerin levels significantly rose in allogeneic-transplanted compared with syngeneic mice, as soon as GvHD signs were manifested (Figure 1A). This finding supports the idea that chemerin levels rise as a part of the ongoing inflammatory response and are crucial for guiding the action of protective cells, including CMKLR1^+^ donor-derived monocytes. In patients, high chemerin levels at early time points after HSCT, which, as in mice, could mirror the molecular inflammatory state of the patient, are a predictor of the possibility of developing GvHD. Although the validation of this result in a larger cohort of patients is required, this evidence further supports the involvement of chemerin in the early immune-mediating events occurring after HSCT and suggests this protein as a potential predictive biomarker to guide patient-specific preemptive therapy to avoid GvHD onset and improve HSCT outcome (65–67). Furthermore, the correlation in HSCT patients of chemerin plasma levels and the number of circulating CMKLR1^+^ monocytes with the GvHD clinical outcome will provide precious clues to understand whether the CMKLR1/chemerin axis could be exploited as a new therapeutic target in HSCT patients experiencing severe GI GvHD.

Despite the important advances in the understanding of its pathogenesis and therapy, GvHD remains a major complication of HSCT. Our study paves the way to a future in-depth investigation of the potential clinical implications of the chemerin/CMKLR1 axis in controlling intestinal inflammation in patients experiencing severe GI GvHD.

## Methods

### Mice.

C57BL/6 (B6, H-2^b^) and BALB/c (H-2^d^) WT mice were purchased from Charles River Laboratories. *Cmklr1*-knockout mice (*Cmklr1*-KO C57BL/6, H-2^d^) were previously described (13).

### Patients and samples.

Seventy-one pediatric patients with hemato-oncological diseases, who underwent allogeneic HSCT at the Pediatric Departments of San Gerardo Hospital, Monza, Italy, were enrolled in the study. Peripheral blood samples for hChemerin level measurement were collected before the start of the conditioning regimen, on day 0 (i.e., at day of HSCT) and weekly after HSCT. Plasma was obtained after centrifuging of whole blood, collected in EDTA-containing Vacutainer tubes (Becton Dickinson), at 1,000*g* for 10 minutes at 4°C. Plasma samples were then frozen at a minimum of –80°C until hChemerin was evaluated by ELISA.

### BMT model using CMKLR1-KO or WT donor cells in a WT recipient.

To induce acute GvHD, BMT experiments were performed as previously described (68). In detail, BALB/c mice were lethally irradiated with 700 cGy (RADGIL, Ghilardoni) in 2 split doses and received 10 × 10^6^ bone marrow cells and 5 × 10^6^ splenocytes harvested from WT mice (C57BL/6) or CMKLR1*-*KO mice. All mice were males 8–12 weeks old. Recipient mice were treated with gentamicin (80 mg/L) (Hospira) 7 days before transplant. Gentamicin was thereafter continued to prevent and treat infections as a consequence of the immunosuppression resulting from the conditioning regimen.

Mice were scored 3 times a week for clinical GvHD using a modified scoring system that includes posture, activity, skin integrity, fur texture, and diarrhea attested by liquid stool production at time of mouse manipulation or its presence at the anal area (69). A score of 0–2 was assigned for each parameter evaluated with a total score of 0–10. At the same time points mice were monitored for weight changes.

### BMT model using WT donor cells in CMKLR1-KO or WT recipients.

Lethally irradiated CMKLR1-KO or -WT C57BL/6 mice (H-2^b^) were intravenously transplanted with 10 × 10^6^ bone marrow cells and 20 × 10^6^ splenocytes obtained from WT BALB/c mice (H-2^d^). All mice were 8-week-old males. Recipient mice were treated with gentamicin (80 mg/L) (Hospira) starting from day 7 before transplant until sacrifice to prevent and treat infections. Mice were weighed and GvHD was scored, as previously described.

### Chemerin ELISA.

Peripheral blood samples of transplanted mice were collected the day before TBI and every 3 days beginning 24 hours after transplantation. Plasma was separated from the cell fraction by centrifugation at 1,000*g* for 10 minutes at 4°C and was cryopreserved before use. Chemerin plasma levels were measured using mouse chemerin ELISAs (DuoSet, R&D Systems) following the manufacturer’s instructions. Human chemerin was further evaluated in the peripheral blood of 81 pediatric patients using human chemerin ELISA (DuoSet, R&D Systems).

### In situ hybridization for Cmklr1^+^ and Rarres2^+^ cells.

To localize *Cmklr1^+^* and *Rarres2^+^* cells, mouse tissues were analyzed with RNAscope assay (Advanced Cell Diagnostics) using RNAscope 2.5 HD Assay-RED kit and Mm-Cmklr1-C2 probe (catalog 509811-C2) recognizing nucleotides 39–950 of the Cmklr1 reference sequence NM_008153.3 or Mm-Rarres2 probe (catalog 572581) recognizing nucleotides 24–520 of the Cmklr1 reference sequence NM_001347167.1. The sections from fixed mouse tissue blocks were treated following the manufacturer’s instructions. Briefly, freshly cut 3 μm sections were deparaffinized in xylene and treated with the peroxidase block solution for 10 minutes at room temperature followed by the retrieval solution for 15 minutes at 98°C and by Protease Plus (ACD Bio) at 40°C for 30 minutes. Control probes included Mm-Polr2a-C2 (catalog 321651) and dapB-C2 (catalog 527659-C2) as positive and negative control, respectively. The hybridization was performed for 2 hours at 40°C. The signal was revealed using RNAscope 2.5 HD Detection Reagent and FAST RED (ACD Bio). Combined RNAscope and immunohistochemistry for IBA1 (polyclonal rabbit, 1:600; Wako) and claudin-5 (rabbit, clone EPR7583, 1:100; Abcam) were used to identify the cellular source of *Cmklr1^+^* and *Rarres2^+^* cells. To this end, the detection by RNAscope was combined with immunoreaction visualized using Mach4 Universal AP-Polymer Kit (Biocare Medical) followed by Ferangi Blue (Biocare Medical).

### Histological analysis (GvHD pathological scoring).

To evaluate GvHD severity, mice were sacrificed at different time points after BMT, and hair and skin from the dorsal region, lung, liver, and intestine (ileum and colon) were fixed in 10% neutral-buffered formalin for at least 48 hours at room temperature, routinely processed for paraffin embedding, sectioned at 4 μm thickness, and stained with H&E. Sections were evaluated in a blinded fashion under a light microscope. GvHD lesions in skin, lung, liver, and small and large intestine were scored semiquantitatively according to the histological GvHD grading system reported in Supplemental Table 1. Large intestine and small intestine total GvHD histological scores (range 0–16) were calculated by addition of the individual scores of colitis, crypt hyperplasia, intraepithelial lymphocytes, and epithelial damage (Supplemental Table 1). Lung histological score (range 0–6) was calculated by addition of the individual scores of periluminal infiltrate and bronchiolar epithelial degeneration (Supplemental Table 1). Skin score, (range 0–24) was calculated by addition of the individual scores of epidermal hyperplasia and hyperkeratosis, vacuolization of basal epidermal cells, and other parameters indicated in Supplemental Table 1. Liver histopathological damage was scored 1–4 (Supplemental Table 1).

### FISH analysis.

Unfixed frozen tissue samples embedded in OCT were cut in slides of 5 μm and fixed in Carnoy’s buffer. Then slides were hybridized with 5 pmol of a universal bacterial 16S fluorescent rRNA probe (EUB338-Cy3, 5′-GCTGCCTCCCGTAGGAGT-3′ Cy5) in a hybridization buffer containing 0.9 M NaCl, 20 mM Tris/HCl (pH 7.3), and 0.01% SDS, overnight in a dark humid chamber at 46°C. Afterward, the slides were counterstained for DNA by DAPI (1:25,000 dilution; Invitrogen) for 10 minutes at room temperature. The probe was synthesized commercially and 5′-end-labeled with fluorochrome Cy3 (Invitrogen), giving a bright orange signal.

To reveal unspecific staining of EUB338-Cy3, hybridization buffer was used as negative control. Representative images were obtained from blind acquisition of 5 fields per animal. The images were acquired by confocal microscopy with a 60 Olympus Plan Achromat Objective.

### Immunohistochemical analysis.

For immunohistochemistry, serial 4 μm formalin-fixed and paraffin-embedded sections from colon at day +21 after BMT were immunostained with primary antibodies raised against CD3ε (M20) (sc-1127, Santa Cruz Biotechnology), CD45R/B220 (RA3-6B2, BD Pharmingen), MPO (A0398, Dako), iNOS (Ab15323, Abcam), and arginase-1 (sc-18354, Santa Cruz Biotechnology).

The sections were labeled by the avidin-biotin-peroxidase procedure with a commercial immunoperoxidase kit (Vectastain Standard Elite, Vector Laboratories). Immunoreactivity was revealed by incubation of the sections with 3,3′-diaminobenzidine (Vector Laboratories). Sections were counterstained with Mayer’s hematoxylin. For each sample, serial sections incubated with a 10% solution of goat or horse normal serum served as negative controls.

To evaluate the extent of inflammatory cells infiltrating the colon, the number of positive cells (CD3ε, CD45R/B220, MPO) or the percentage positive area (arginase-1, iNOS) was evaluated using the NIH ImageJ analysis program (http://rsb.info.nih.gov/ij/) in 3 ×400 hot spot microscopic fields, and the results were normalized on the examined intestinal mucosa area.

### Flow cytometry.

To obtain a single-cell suspension, large intestine was firstly digested for 2 minutes in a 1 mM DTT (Roche) solution, then cut into smaller pieces and digested using a 16 μg/mL DNase I (Roche) and 0.4 mg/mL collagenase II (Gibco) enzymatic solution for 2 hours at 37°C. Total nucleated cells were stained with the following antibodies: PE-Cy7–anti–mouse CD45 (30F11, eBioscience), PerCP-Cy5.5–anti–mouse CD11b (clone M1/70, eBioscience), eFluor450–anti–mouse Ly6C (HK1.4, eBioscience), APC–anti–mouse Ly6G (RB6-8C5, eBioscience), Alexa Fluor 488–anti–mouse F4/80 (BM8, eBioscience), APC–eFluor 780–anti–mouse B220 (RA3-6B2, eBioscience), and APC–anti–mouse CD3ε (145-2C11, eBioscience) and LIVE/DEAD Fixable Aqua Dead Cell Stain Kit (Life Technologies). After staining, cells were fixed using PBS with 1% paraformaldehyde. Samples were acquired using a Canto II Flow Cytometer (BD Biosciences) and analyzed using FACSDiva Software (BD Biosciences).

### Adoptive transfer of monocytes and pDCs.

To perform monocyte adoptive transfer, splenocytes and bone marrow cells were collected from both C57BL/6 WT and CMKLR1-KO mice. Monocytes were purified by negative selection using the mouse Monocyte Isolation kit (Miltenyi Biotec). The purity of monocytes, evaluated by flow cytometry as percentage of CD11b^+^Ly6C^+^ cells on gated CD45^+^ cells, was greater than 90%. After conditioning regimen, 0.2 × 10^6^ monocytes were infused together with bone marrow cells and splenocytes (10 × 10^6^ bone marrow cells and 5 × 10^6^ splenocytes). The number of monocytes was calculated based on the quantity of cells collected from a bone marrow and splenocyte cell mix of 15 × 10^6^ using the above-mentioned isolation kit. This number was further confirmed by flow cytometry analyses evaluating the percentage of CD45^+^Ly6G^–^Ly6C^hi^ monocytes present in the bone marrow and splenocyte cell mix (data not shown). pDCs were isolated from the spleen of C57BL/6 WT mice by means of Plasmacytoid Dendritic Cell Isolation Kit II (Miltenyi Biotec). Indeed, 0.5 × 10^6^ pDCs were coinjected together with 10 × 10^6^ bone marrow cells and 5 × 10^6^ splenocytes collected from C57BL/6 WT or CMKLR1-KO mice in lethally irradiated BALB/c mice.

### DSS-induced colitis model.

Colitis was induced in 6- to 8-week-old female C57BL/6 mice by administration of 3% DSS in drinking water ad libitum for 8 days. Body weight and disease activity index were evaluated daily as previously described (70). At day 8, mice were sacrificed, colon length was measured, and colons were Swiss-rolled, fixed in 4% formalin for 24 hours, paraffin-embedded, and sectioned (4 μm). H&E staining was performed to evaluate tissue and cell morphology, which was scored by a pathologist under a blinded protocol as described previously (70).

### In vivo intestinal permeability assay.

The gut barrier function was evaluated by in vivo intestinal permeability using FITC-dextran (MW 4000; FD4, Sigma-Aldrich) (71). Briefly, FD4 was orally administered to mice (4 mg/10 g body weight). Mice were fasted for 4 hours prior to sacrifice. FD4 serum concentration was determined on a fluorescence plate reader (excitation, 485 nm; emission, 528 nm; SINERGY H4, Biotek).

### RNA extraction from paraffin-embedded or OCT-embedded mouse colon sections.

RNA was extracted for whole-transcriptome analyses from both formalin-fixed, paraffin-embedded (FFPE) and OCT–snap-frozen colon tissues harvested at different time points from GvHD mice. In detail, FFPE blocks were sectioned to prepare 5 μm sections (10 for each mouse) on glass slides for RNA extraction. Colon tissue was scraped from slides by means of a sterile blade and collected into a centrifuge tube. Extraction of total mRNA was performed using Maxwell 16 LEV RNA FFPE Purification Kit (Promega) according to the manufacturer’s protocol and a separate automated Maxwell 16 instrument (Promega). In the case of OCT-embedded tissues, frozen blocks were sectioned by means of a cryostat to prepare 10-μm-thick sections (10 for each mouse) that were collected into a centrifuge tube containing RNeasy Lysis buffer (RLT, Qiagen). RNA was then extracted using RNeasy Mini Kit (Qiagen) following the protocol “Purification of total RNA from animal tissue.” RNA obtained from FFPE and OCT-embedded tissues was quantified using Qubit RNA HS Kit on a Qubit 4 Fluorometer (Thermo Fisher Scientific).

### Whole-transcriptome analysis by next-generation sequencing of mouse colon tissue.

Whole-transcriptome RNA-sequencing (RNA-Seq) analysis was performed on mouse colon tissue by next-generation sequencing, using the Universal RNA-Seq kit (NuGen, Tecan). The Universal RNA-Seq is an end-to-end solution for strand-specific RNA-Seq library construction, using 250 ng amounts of total RNA obtained from mouse colon tissue in the case of FFPE sections and 400 ng in the case of OCT-embedded sections. The workflow consists of double-stranded cDNA generation using a mixture of random priming, optional fragmentation of double-stranded cDNA, end repair, adaptor ligation, strand selection, targeted transcript depletion with AnyDeplete (Tecan) for globin genes, and PCR amplification to produce the final library. The yields of final libraries were assessed by Qubit 4.0 fluorimeter, and their sizes were assessed by Agilent Bioanalyzer. The libraries were analyzed by paired-end sequencing on NextSeq550 Illumina platform, 2×75, sequencing 12 samples on a High Output v2.5 150-cycle cartridge. FASTQ files are available in the ArrayExpress database (www.ebi.ac.uk/arrayexpress) under accession numbers E-MTAB-12231 and E-MTAB-12300. Raw FASTQ sequences were quality-tested with FastQC (https://www.bioinformatics.babraham.ac.uk/projects/fastqc/) and aligned against the GRCh38/hg38 reference human genome with the splice-aware aligner Star v2.7.9 (https://github.com/alexdobin/STAR; commit ID f4fa8e8), using the quantMode GeneCounts parameter for read count generation. Binary alignment files were subsequently indexed with Samtools (72). The Bioconductor package DESeq2 v1.30 (73) was used to perform differential gene expression analysis. Sorted, indexed BAM alignment files were used for manual data inspection using the Integrative Genomics Viewer (74). GSEAs were performed with GSEA software v4.2.1 (https://www.gsea-msigdb.org/gsea/downloads.jsp) using a gene set permutation scheme with 1,000 random permutations. GSEA was applied on global protein-coding gene expression profiles: significant gene sets were selected based on nominal *P* value less than 0.05 and FDR less than 0.25.

### Statistics.

Data were represented graphically separately by group of interest. For numerical variables observed repeatedly in time, 2 types of graphs were used. The graphs of means displayed across time were obtained by connecting of points of contiguous times with segments. Dot plots were obtained using jitter points to avoid overplotting of tied data and displaying the medians (horizontal line). Numerical variables observed at a single time point were all represented by dot plots obtained using jitter points to avoid overplotting of tied data and displaying the medians (horizontal line) or box plots with whiskers (minimum to maximum) and displaying the medians (horizontal line) and the mean (plus symbol). A Kaplan-Meier estimate of the survival (expressed as percentage of survivors), adjusting for the presence of right censoring, was performed.

Two-sided Mann-Whitney test or unpaired 2-tailed *t* test was used for comparisons between groups at fixed time points, as described in the figure legends. For testing on numerical variables observed repeatedly in time, we used Mann-Whitney test or unpaired 2-tailed *t* test with Bonferroni-Dunn correction for multiple comparison, as described in the figure legends. Survival curves were compared using the log-rank test.

Variation of human chemerin levels across visits was modeled using linear mixed models with random intercepts. Chemerin values were transformed on log scale before modeling. Results are reported as estimated fold change and corresponding 95% CI. Occurrence of GvHD during the 100 days after HSCT was modeled as a function of percentage variation of chemerin between time 0 and baseline [computed as (baseline – time 0)/baseline] using a proportional hazard Cox model, both with and without accounting for regimen. Results are reported as estimated hazard ratio and corresponding 95% CI.

### Study approval.

Experimental protocols for animal studies were approved by the Ethics Committee for Animal Experimentation of both Ministero della Salute and University of Milano-Bicocca (approvals 25/2014-B, issued on January 28, 2014, and 1092/2016-PR, issued on June 7, 2016).

The analysis of chemerin levels in plasma samples collected from HSCT patients was approved by the Institutional Review Board (AIEOP-BFM ALL 2009 protocol; EudraCT-2007-004270-43); written informed consent from either parents or legal guardians was obtained.

## Author contributions

ED performed research, interpreted the data, and wrote the manuscript. PV performed experiments, collected patient samples, and critically revised the manuscript. SV and AP evaluated the susceptibility of *Cmklr1*-KO mice to acute DSS-induced colitis and performed FISH analysis regarding bacteria translocations. CR and LB performed immunohistochemistry staining and evaluated results. RP and GF performed whole-transcriptome experiments and analyzed and interpreted the data. MB and WV performed RNAscope experiments in different GvHD target organs and interpreted the data. DB, FS, and ADP performed in vivo experiments and critically revised the manuscript. SB provided clinical information about transplanted patients. ET and MGV performed statistical analyses for in vivo experiments. SC performed statistical analyses concerning the correlation of chemerin plasma levels and onset of acute GvHD in HSCT patients. AB critically revised the paper. SS contributed to the design of the study, interpreted the data, and contributed to manuscript preparation. GD designed the study, supervised the research, interpreted the data, provided funds, and critically revised the manuscript.

## Supplementary Material

Supplemental data

## Figures and Tables

**Table 3:**
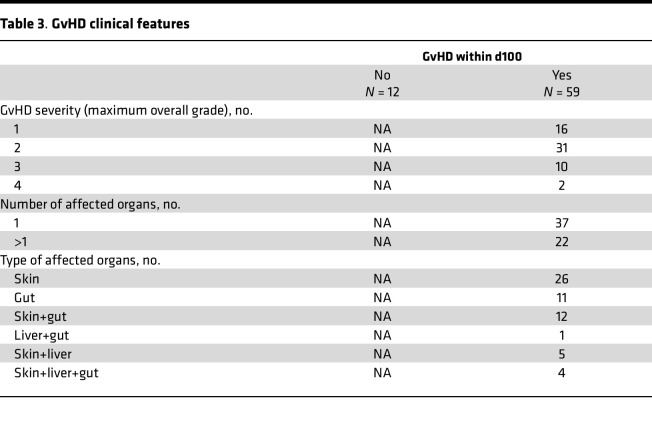
GvHD clinical features

**Table 2:**
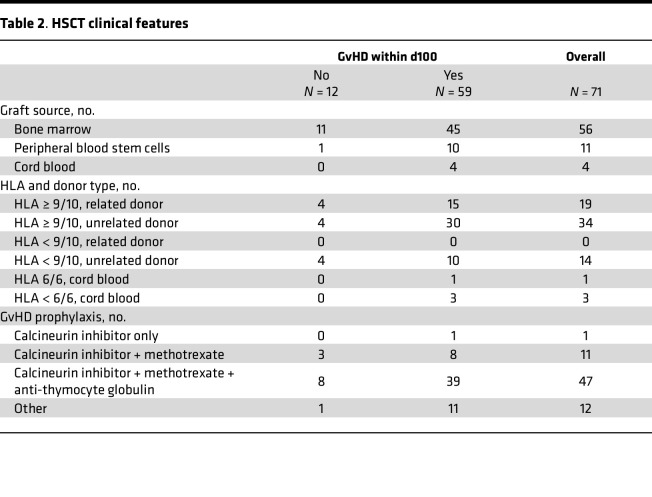
HSCT clinical features

**Table 1:**
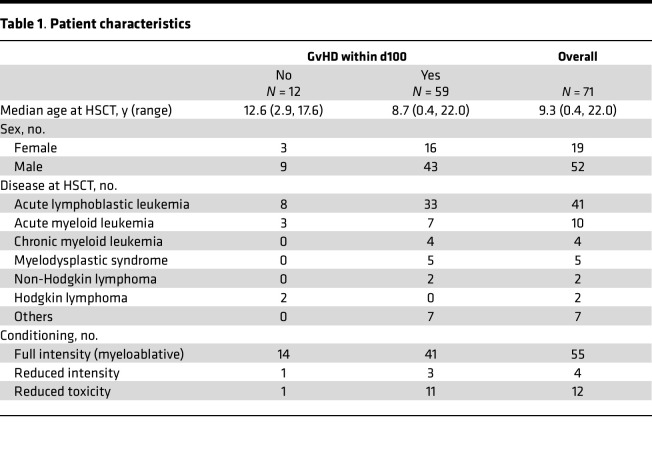
Patient characteristics

## Version 1 03/08/2023

Electronic publication
