# Supplementary material for: The chemerin/CMKLR1 axis regulates intestinal graft-versus-host disease
Source: JCI Insight. 2023 Mar 8;8(5):e154440. doi: 10.1172/jci.insight.154440 (PMC10077469; doi:10.1172/jci.insight.154440)
Supplement: Supplemental data [file jciinsight-8-154440-s055.pdf]

Supplementary figures

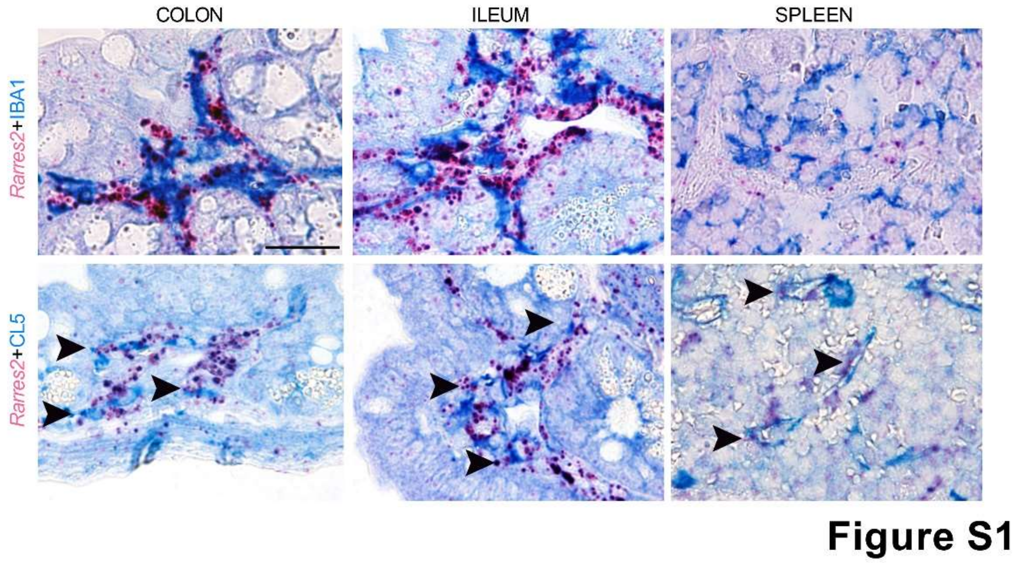

Figure S1. RNA scope analysis of *Rarres2* mRNA in combination with IBA1 and CL5 immunohistochemical staining in syngeneic-transplanted control mice.

Lethally irradiated BALB/c mice were transplanted with bone marrow and splenocytes obtained from BALB/c mice, as syngeneic control. The identity of chemerin producing cells was investigated by RNA scope staining of *Rarres2* mRNA. *Rarres2* staining (red signal) was observed in IBA1^{+} macrophages (immunohistochemistry, blue) and in Claudin (CL)5^{+} endothelial cells (immunohistochemistry, blue; arrow heads= CL5^{+} endothelial cells). Magnification: 600x, scale bar 33 micron.

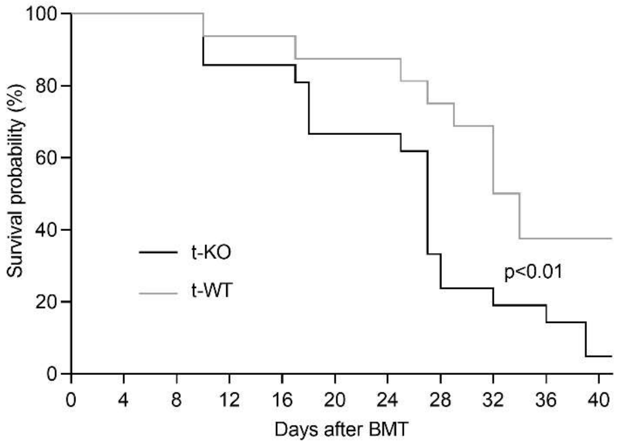

Figure S2

Figure S2. Long-term evaluation of the survival of mice transplanted with CMKLR1-KO or -WT donor cells.

Lethally irradiated BALB/c (H-2^d) mice were transplanted with bone marrow and splenocytes obtained from C57BL/6 CMKLR1$^{-/-}$ mice (t-KO, B6, H-2^b) or C57BL/6 wild type mice (t-WT, H-2^b). Survival curves of t-WT (n=21) and t-KO mice (n=16), until day +41 after transplant. P was calculated by Long-rank (Mantel-Cox) test.

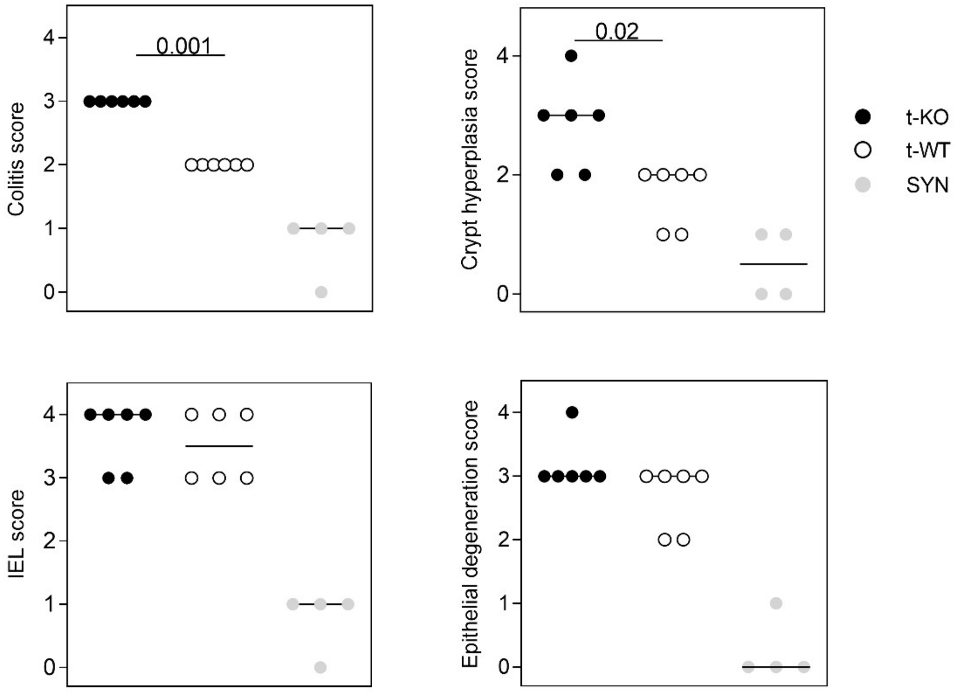

Figure S3

Figure S3. Histopathological characterization of the large intestine GvHD damage in mice transplanted with CMKLR1-KO donor cells.

Lethally irradiated BALB/c (H-2^d) mice were transplanted with bone marrow and splenocytes obtained from C57BL/6 CMKLR1$^{-/-}$ mice (t-KO, B6, H-2^b), C57BL/6 wild type mice (t-WT, H-2^b) or BALB/c mice (H-2^d, syngeneic control-SYN). Large intestine was harvested 21 days after BMT. H&E staining was used to perform histopathological analysis. Colitis, crypt hyperplasia, intraepithelial lymphocytes (IEL) and epithelial damage received a score between 0 and 4 based on severity (see **Table S1**). Data are shown as dot plots with median values (horizontal lines); t-KO (n=6); t-WT (n=6); SYN (n=4). P-values: Mann-Whitney test.

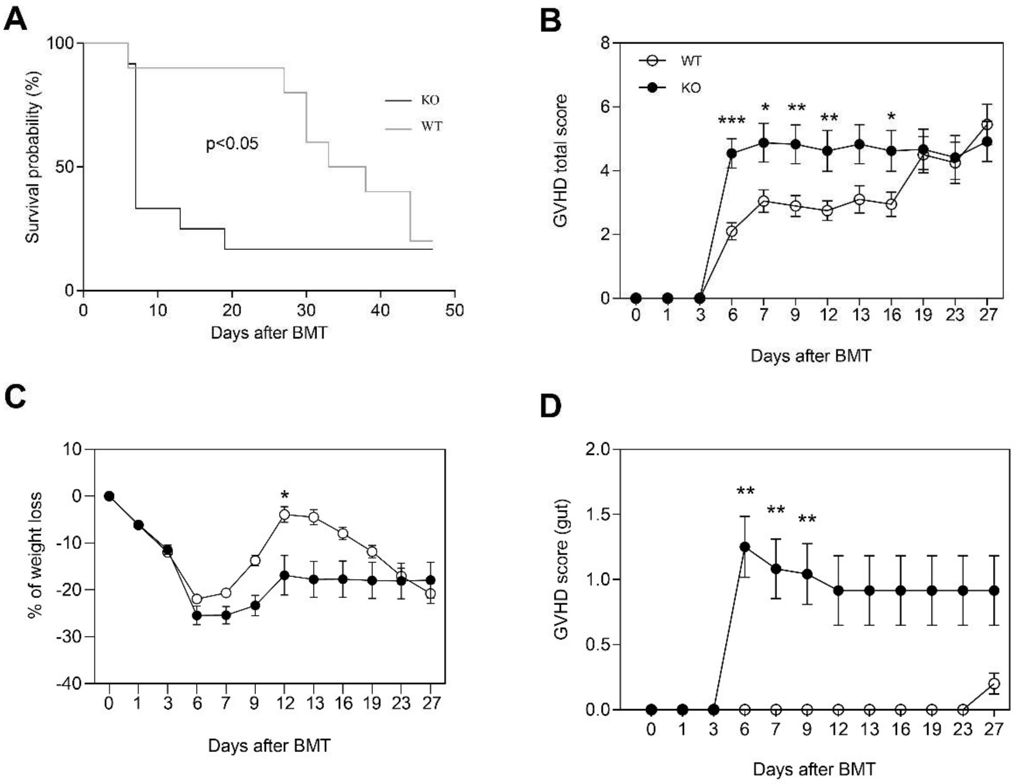

Figure S4

Figure S4. CMKLR1-KO recipient mice transplanted with WT donor cells developed a severe GI GvHD.

Lethally irradiated C57BL/6 (H-2b) WT or CMKLR1$^{-/-}$ (KO) mice were transplanted with $10x10^6$ bone marrow and $20x10^6$ splenocytes obtained from BALB/c mice (H-2d). (A) The graph shows the survival curves of KO mice (n=12) compared to WT mice (n=10) of one experiment. *p<0.05, Gehan-Breslow-Wilcoxon test. (B) The graph shows acute GvHD total score. In addition, the percentage of weight loss (C) and the gut GvHD score (D) are illustrated. Data are shown as mean ± SEM. * Adjusted P value <0.05, ** adjusted P value <0.01, *** adjusted P value <0.001, Mann-Whitney test with Bonferroni-Dunn's correction for multiple comparisons.

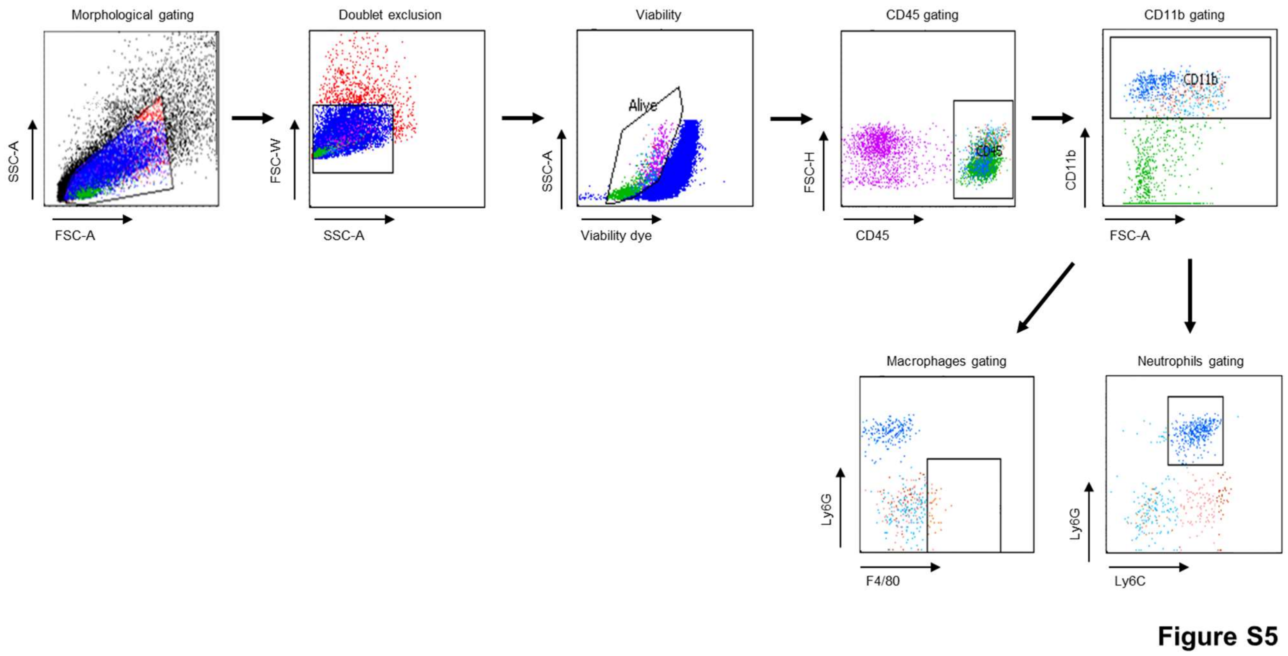

Figure S5. Gating strategy for the flow cytometry evaluation of macrophages and neutrophils in the large intestine of transplanted mice.

The percentage of macrophages was evaluated by analysing Ly6G and F4/80 expression (Ly6G^{-} F4/80^{+}) on gated CD45^{+}CD11b^{+} alive large intestine cells, as shown in the dot plots. The percentage of neutrophils was evaluated by analysing Ly6G and Ly6C expression (Ly6G^{+}Ly6C^{int}) on gated CD45^{+}CD11b^{+} alive large intestine cells.

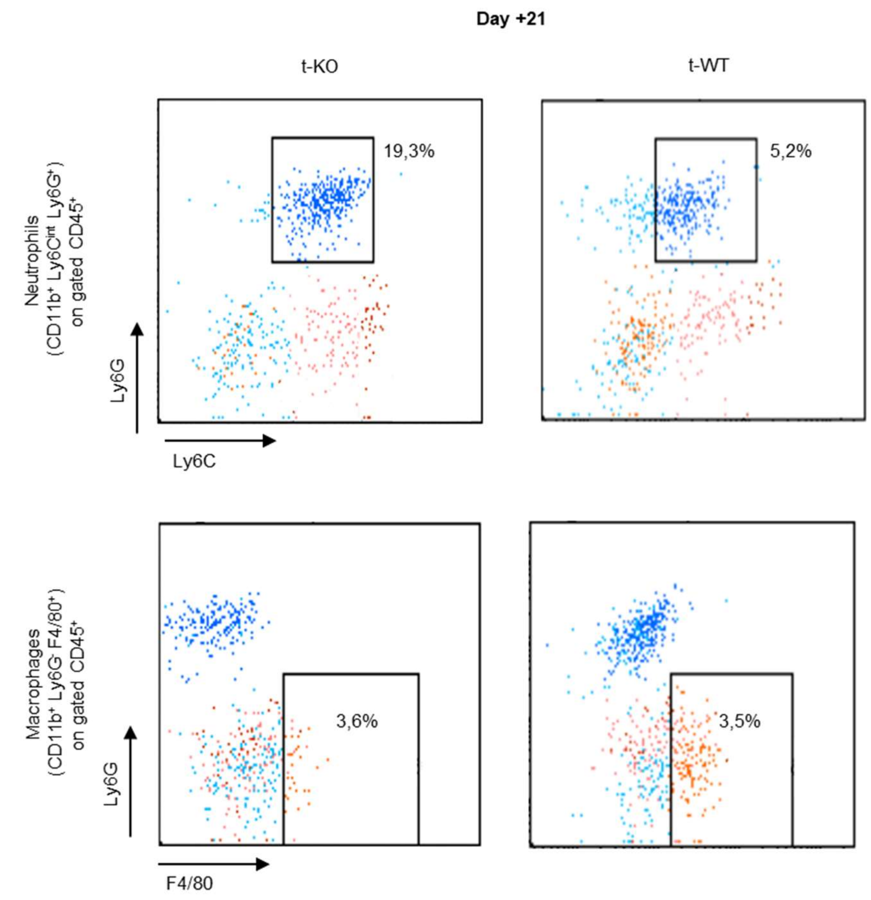

Figure S6

Figure S6. Flow cytometry evaluation of macrophages and neutrophils in the large intestine of mice transplanted with CMKLR1-KO donor cells.

Lethally irradiated BALB/c (H-2^d) mice were transplanted with bone marrow and splenocytes obtained from C57BL/6 CMKLR$1^{-/-}$ mice (t-KO, B6, H-2^b) or C57BL/6 wild type mice (t-WT, H-2^b). Large intestine was harvested and the percentage of macrophages ($CD45^+CD11b^+Ly6G^-F4/80^+$) and neutrophils ($CD45^+CD11b^+Ly6C^{int}Ly6G^+$) was evaluated at day +21 after BMT. Representative flow cytometry dot plots for both t-KO and t-WT mice are shown in the figure.

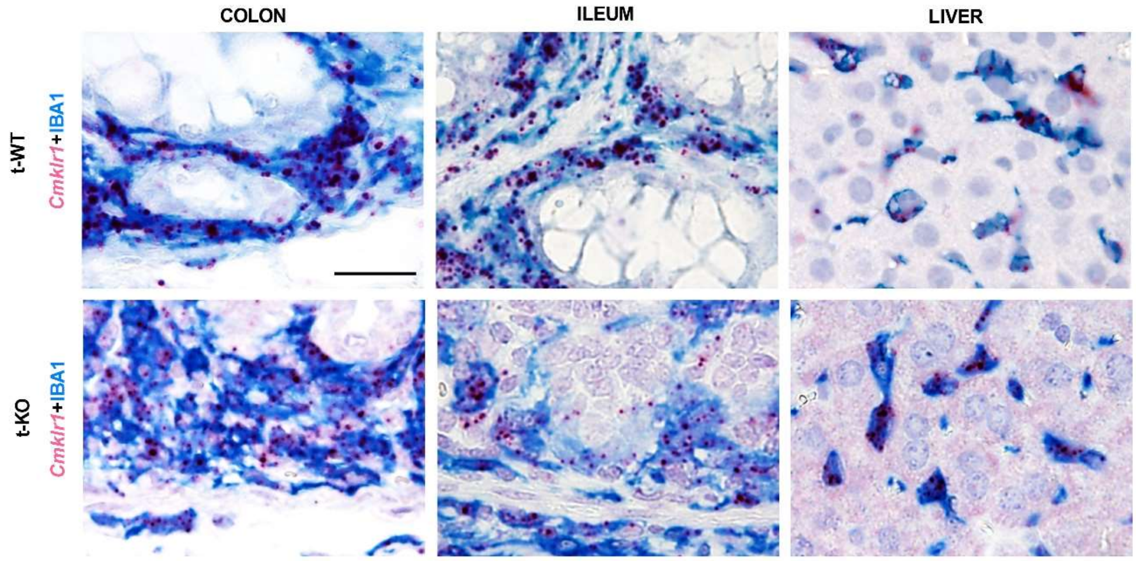

Figure S7

Figure S7. Analysis of *Cmklr1*$^+$ IBA1$^+$ macrophages in mouse intestine and liver.

Cmklr1 mRNA expression (red signal) was investigated by RNA scope staining on IBA1$^+$macrophages (immunohistochemistry, blue) in mouse colon, ileum and liver from t-WT and t-KO mice. Magnification: 600x, scale bar 33 micron.

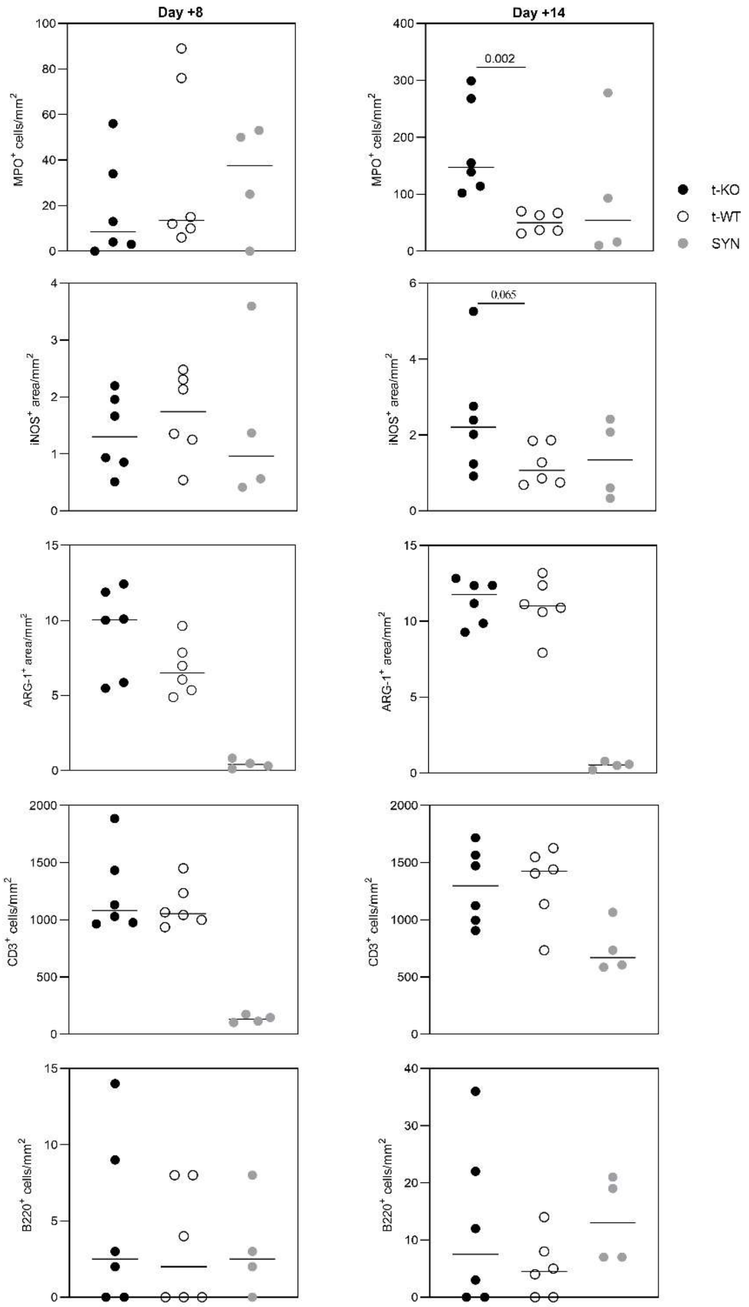

Figure S8

Figure S8. Characterization of the immune infiltrate in the large intestine of mice transplanted with CMKLR1-KO donor cells at early disease stages.

Lethally irradiated BALB/c (H-2^d) mice were transplanted with bone marrow and splenocytes obtained from C57BL/6 CMKLR1$^{-/-}$ mice (t-KO, B6, H-2^b), C57BL/6 wild type mice (t-WT, H-2^b) or BALB/c mice (SYN, H-2^d). Large intestine was harvested +8 and +14 days after BMT. MPO, iNOS, Arg-1, CD3 and B220 expression were studied by immunohistochemistry to evaluate immune cell infiltration in colon mucosa. Antigen quantification as number of positive cells/mm^2 or immunoreactive area/mm^2 of colon mucosa are shown for t-KO (n=6), t-WT (n=6), and SYN (n=4). Data are represented as dot plots, the black bar indicates the median. P-values: Mann-Whitney test.

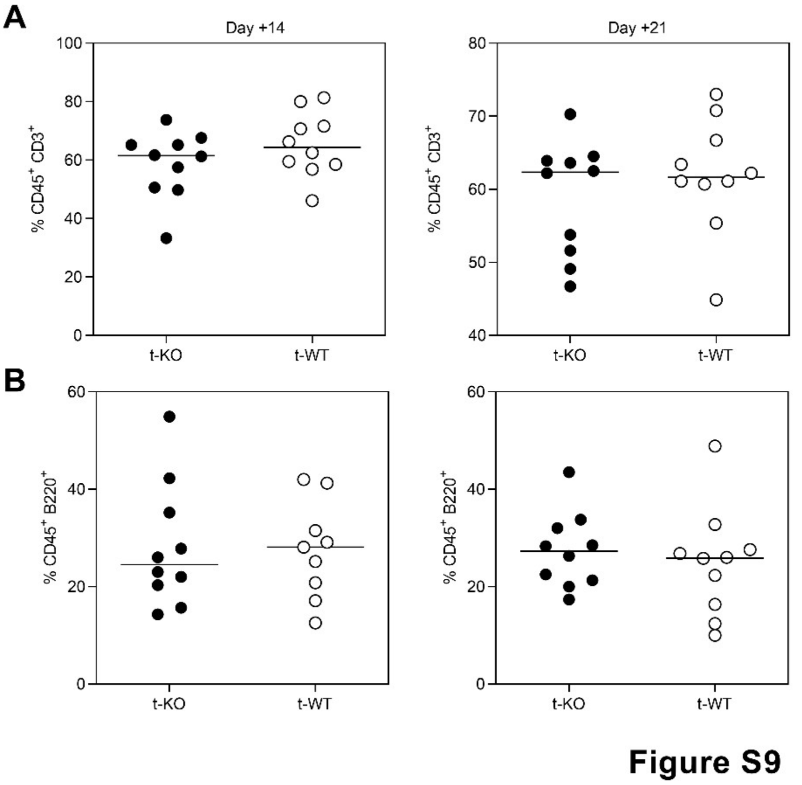

Figure S9. Flow cytometry analyses of CD3 and B220-expressing cells in the colon of mice transplanted with CMKLR1-KO donor cells.

Lethally irradiated BALB/c (H-2^d) mice were transplanted with bone marrow and splenocytes obtained from C57BL/6 CMKLR1$^{-/-}$ mice (t-KO, B6, H-2^b) or C57BL/6 wild type mice (t-WT, H-2^b). (A) Large intestine was harvested and the percentage of CD3+ cells was evaluated at day +14 and +21 after BMT. (B) The percentage of B220+ cells was evaluated at day +14 and +21 after BMT. t-KO (n=10); t-WT (n=10) for CD3 staining and t-KO (n=10); t-WT (n=9) for B220 staining. P-values: Mann-Whitney test.

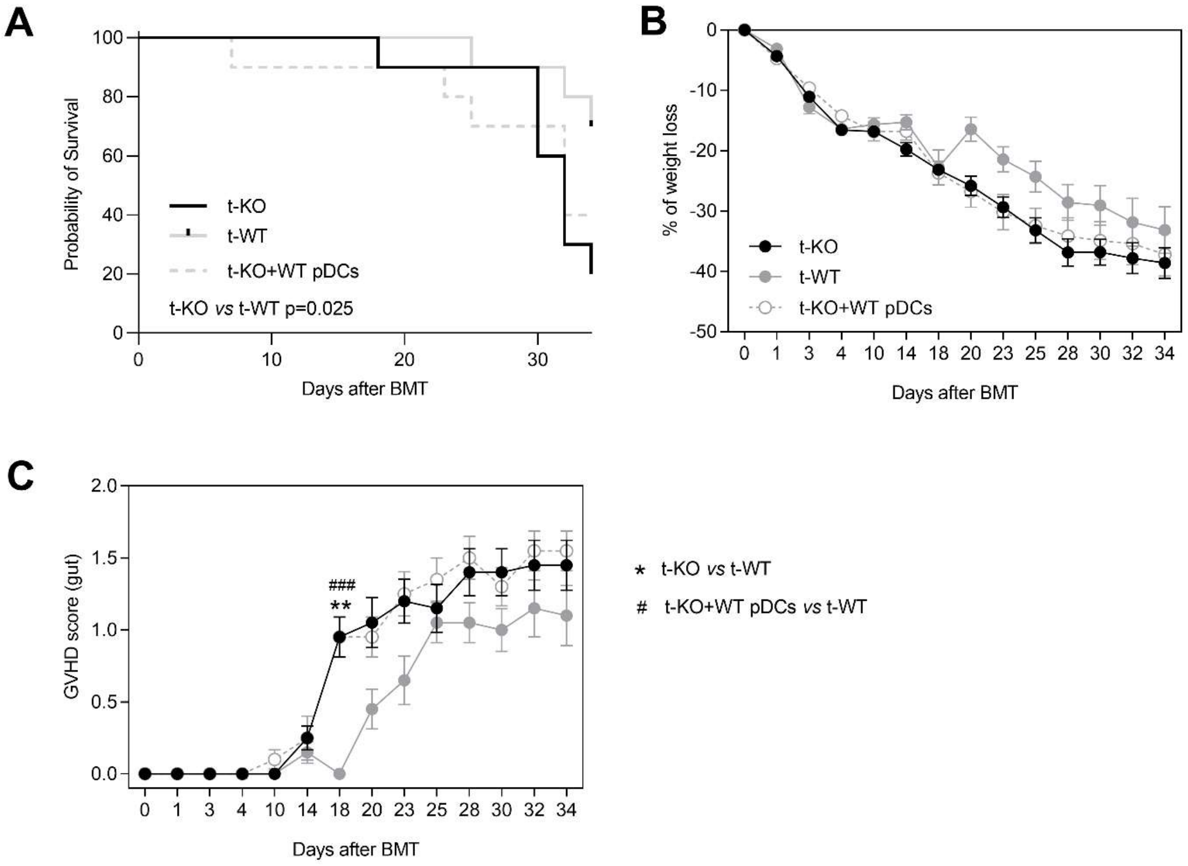

Figure S10

Figure S10. The adoptive transfer of CMKLR1-expressing plasmacytoid dendritic cells (pDCs) in KO-transplanted mice did not improve gastrointestinal (GI)-GvHD.

Lethally irradiated BALB/c (H-2^d) mice were transplanted with bone marrow and splenocytes obtained from C57BL/6 wild type mice (t-WT, H-2^b) or CMKLR1$^{-/-}$ mice (t-KO, B6, H-2^b), in combination or not with purified WT pDCs (t-KO+WT pDC). **(A)** Survival curves of t-WT mice and t-KO mice added or not with WT pDCs. t-KO (n=10); t-WT (n=10), t-KO+WT pDCs (n=10). P was calculated by Long-rank (Mantel-Cox) test. (B) Mice were weighted every 2-3 days to evaluate the involvement of the GI tract and observed for the presence of diarrhoea (C). Data are shown as mean ± SEM. Data are from one explorative experiment, t-KO (n=10); t-WT (n=10); t-KO+WT pDCs (n=10). * Adjusted P value <0.05: t-KO versus t-KO+WT pDCs, as evaluated by Mann-Whitney test with Bonferroni-Dunn’s correction for multiple comparisons.

Table S1: GvHD histopathological score

Skin

Score	Epidermal hyperplasia and hyperkeratosis	Vacuolization of basal epidermal cells	Intra-epithelial lymphocytes (IEL)	Apoptotic cells	Pustules/Erosions/ Ulcers	Lymphohistiocytic Dermatitis
0	absent (1-2 layers of keratinocytes)	absent	absent	absent	absent	absent
1	minimal (focal 3-4 layers of keratinocytes)	minimal	rare (1-2 L in few 400x fields	rare	focal pustules	mild
2	mild (multifocal to Diffuse 3-4 layers of keratinocytes)	mild	occasional (2-5 L in some 400x fields)	occasional	multifocal pustules and/or erosions	moderate
3	moderate (multifocal to diffuse 4-6 layers of keratinocytes)	moderate	multifocal (6-10 L in most fileds)	multifocal	pustules, erosions, and/or ulcers	Marked
4	marked (multifocal to diffuse more than 6 layers of keratinocytes)	marked	diffuse (more than 10 L in most 400x fields)	diffuse	\	\

Total skin score = Epidermal hyperpasia and hyperkeratosis + Vacuolization of basal epidermal cells + IEL + Apoptotic cells + Pustules/erosions/ulcers + Lymphohistiocytic dermatitis

Liver

Score	Description
0	absent or minimal periductal lymphoid cell infiltrate (<5 cells)
1	minimal to mild periductal lymphoid cell infiltrate (< 20 cells) (+/- intraepithelial lymphocytes)
2	mild to moderate periductal lymphoid cell infiltrate (20-40 cells), occasional intraepithelial lymphocytes
3	moderate to marked lymphoid cell infiltrate (> 40 cells), increased intraepithelial lymphocytes, occasional degeneration of biliary epithelial cells, occasional hepatocellular single cell necrosis
4	marked lymphoid cell infiltrate (>40 cells), infiltrating surrounding parenchyma and with occasional bridging of portal zones, increased intraepithelial lymphocytes, increased degeneration of biliary epithelial cells, multifocal hepatocellular single cell necrosis

Lung

Score	Periluminal infiltrates	Bronchiolar epithelial degeneration
0	no infiltrates	absent
1	1-3 cell thick	rare cells (1-2/bronchiole)
2	3-5 cell thick	few cells (2-4/bronchiole)
3	>6 cell thick	moderate no. of cells (>5/bronchiole)

Total lung score = Periluminar infiltrates + Bronchiolar epithelial degeneration

Small intestine

Score	Epithelial degeneration	Intra-epithelial lymphocytes (IEL)	Crypt hyperplasia/atrophy/ dysplasia	Enteritis
0	absent	absent	absent	absent
1	rare crypt epith cell deg	rare IEL (few crypts with 1-2 IEL)	mild hyperplasia	minimal
2	small numbers of deg epith cells in some crypts	small numbers 1-3 of IEL in some crypts	moderate hyperplasia	mild
3	most crypts with small to moderate numbers of deg epith cells, occasional crypt abscesses	small to moderate numbers 1-5 of IEL in most crypts	severe hyperplasia	moderate
4	most crypts with moderate numbers of deg epith cells, numerous crypt abscesses, erosion/ulcer may be present	moderate to large numbers > 5 of IEL in most crypts	dysplasia/atrophy	marked

Total small intestinal score = epithelial degeneration + IEL + crypt hyperplasia + Enteritis

Large intestine

Score	Epithelial degeneration	Intra-epithelial lymphocytes IEL)	Crypt hyperplasia/atrophy/dysplasia	Enteritis
0	absent	absent	absent	absent
1	rare crypt epith cell deg	rare IEL (few crypts with 1-2 IEL)	mild hyperplasia (multifocal mitoses, but well preserved goblet cells)	minimal
2	small numbers of deg epith cells in some crypts	small numbers 1-3 of IEL in some crypts	moderate hyperplasia (increased mitoses, increased length of crypts, rare goblet cells)	mild
3	most crypts with small to moderate numbers of deg epith cells, occasional crypt abscesses	small to moderate numbers 1-5 of IEL in most crypts	severe hyperplasia (increased mitoses, increased length, no goblet cells	moderate
4	most crypts with moderate numbers of deg epith cells, numerous crypt abscesses, erosion/ulcer may be present	moderate to large numbers > 5 of IEL in most crypts	dysplasia/atrophy	marked

Total large intestinal score = epithelial degeneration + IEL + crypt hyperplasia + Enteritis
